# Long noncoding RNA CERS6-AS1 modulates glucose metabolism and tumor progression in hepatocellular carcinoma by promoting the MDM2/p53 signaling pathway

**DOI:** 10.1038/s41420-022-01150-x

**Published:** 2022-08-04

**Authors:** Bo Xu, Yonggang Wei, Fei Liu, Lian Li, Siqi Zhou, Yufu Peng, Bo Li

**Affiliations:** 1 Department of Liver Surgery and Liver Transplantation Center, West China Hospital of Sichuan University, Chengdu, Sichuan China; 2 Department of Hepatic-Biliary-Pancreatic Surgery, The First People’s Hospital of Neijiang, Neijiang, Sichuan China

**Keywords:** Cancer metabolism, Long non-coding RNAs, Cell growth, Cell migration

## Abstract

Hepatocellular carcinoma (HCC) is one of the most serious malignant cancers and has a high fatality rate. However, clinical strategies for the effective treatment of HCC remain lacking. Long non-coding RNAs (lncRNAs) with aberrant expression have been closely correlated with the occurrence and development of HCC. Here we investigated the underlying mechanism of the lncRNA CERS6-AS1 in HCC progression. The expression and prognosis of CERS6-AS1 in HCC patients was explored using The Cancer Genome Atlas. PCR analysis was utilized to measure the expression of CERS6-AS1 in tissues and cell lines. Transwell, wound healing, proliferation and glycolysis assays were conducted to evaluate the function of CERS6-AS1 on HCC cell functions. Bioinformation methods and luciferase assays were used to screen and verify potential target miRNAs and genes. A subcutaneous tumorigenesis model was constructed in nude mice to assess the effect of CERS6-AS1 on tumorigenesis in vivo. CERS6-AS1 was highly expressed in HCC tissues and cell lines. Upregulated CERS6-AS1 expression was remarkably correlated with poor prognosis of HCC patients. High CERS6-AS1 expression facilitated cell growth, invasion and glycolysis of HCC cells. Bioinformatics analyses combining with PCR analysis identified miR-30b-3p as the potential target of CERS6-AS1, and MDM2 mRNA was verified as the target of miR-30b-3p. The expression of miR-30b-3p was negatively correlated with CERS6-AS1, whereas MDM2 was positively associated with CERS6-AS1. Mechanistic studies showed that CERS6-AS1 may sponge miR-30b-3p to elevate MDM2, thus promoting the MDM2-mediated ubiquitin-dependent degradation of the p53 tumor suppressor. MDM2 overexpression or miR-30b-3p inhibitors blocked the inhibitory effect of CERS6-AS1 knockdown on proliferation, migration and glycolysis. CERS6-AS1 depletion reduced tumor formation in the in vivo mouse model. The CERS6-AS1/miR-30b-3p/MDM2/p53 signaling axis may play key roles in regulating HCC progression. CERS6-AS1 may exert as a novel biomarker or therapeutic target for HCC.

## Introduction

Hepatocellular carcinoma (HCC) is one of most fatal solid tumors of the digestive system. HCC is the most common type of liver cancer, accounting for about 85–90% of liver cancer [1]. Unlike alcohol in Europe and the United States, hepatitis B virus infection is the main cause of HCC in China, which accounts for half of all new cases and deaths worldwide [2]. Significant advances in diagnostic and therapeutic strategies for HCC have been achieved in the past years. However, the overall survival of HCC patients is still dismal, and the prognosis of patients is generally poor, with a 5-year survival rate of <20% [3]. Accordingly, it is crucial to better comprehend the detailed molecular mechanism of HCC pathogenesis and progression, and to identify effective biomarkers and therapeutic targets that are valuable for HCC diagnosis and prognosis.

The pathogenesis of HCC is a complex process involving genetic and epigenetic changes [4]. Accumulating evidence has shown lncRNAs play multiple roles in the pathogenesis of liver cancer [5], with roles in angiogenesis, tumorigenesis, apoptosis, signal transduction, and immune evasion [6]. Long non-coding RNAs (lncRNAs), which are mainly composed of more than 200 nucleotides and lack the ability to directly encode proteins, are a class of non-coding RNAs and involved in a variety of biological regulation [7]. It is worth noting that lncRNAs locating in the cytoplasm have been shown to function as competing endogenous RNA (ceRNA), in which lncRNAs bind and “sponge” miRNAs, thereby regulating target mRNAs [8]. Recent research has illustrated lncRNAs are involved in the regulation of HCC progression. For instance, lncRNA SNHG7 enhances the cell growth and metastasis of HCC by enforcing FOXK2 via binding to miR-122-5p [9]. LncRNA DLX6-AS1 promotes HCC progression by sponging miR-513c to block its inhibitory effect on the target Cullin4A gene, promoting the ubiquitination-mediated degradation of ANXA10 and leading to increased malignant phenotypes of HCC cells [10]. LncRNAs can also directly bind proteins to regulate stabilization and thereby enhancing biological function. For example, lncRNA RP11-286H15.1 suppresses HCC progression by binding PABPC4 and promoting ubiquitination of PABPC4 [3].

LncRNAs may also play a role in tumorigenesis through reprogramming glucose metabolism, which is an important feature of cancer cells [11]. Unlike normal cells, cancer cells metabolize glucose through glycolysis, enhancing glucose uptake and lactic acid production. This phenomenon, known as the Warburg effect, gives cancer cells a great advantage in tumor growth, anti-apoptosis, metastasis and immune escape [12]. The tumor hypoxic microenvironment can induce increased expression of c-Myc in tumor cells, facilitating aerobic glycolysis by enhancing c-Myc-mediated transcriptional regulation of key glycolysis genes, such as PKM2, GLUT1 and HK2 genes [13]. The tumor suppressor p53 is involved in controlling cell metabolism by enhancing mitochondrial aerobic respiration and reducing glycolysis [14]. Recent research has demonstrated that several lncRNAs can modulate the level of glycolysis-related genes or activate signaling pathways to regulate glucose metabolism [11].

Previous studies have indicated that CERS6-AS1 participates in regulating breast cancer and pancreatic cancer progression and influence disease outcome [14–17]. However, the roles of CERS6-AS1 in HCC was still unknown. Thus, we try to uncover the potential function in HCC. In our study, we demonstrate that CERS6-AS1 overexpression is correlated with dismal prognosis in HCC patients. High CERS6-AS1 expression accelerates HCC cell growth, migration and aerobic glycolysis. Mechanistically, CERS6-AS1 binds to miR-30b-3p to repress its suppression on MDM2, thereby promoting MDM2 expression and facilitating ubiquitination-mediated degradation of p53. Above results indicate the CERS6-AS1/miR-30b-3p/MDM2/p53 signaling axis may become effective therapeutic targets for HCC patients.

## Results

### CERS6-AS1 is upregulated in HCC tissues and cell lines and significantly correlates with unfavorable prognosis

First, we analyzed some basic features of CERS6-AS1, including genome location, protein coding potential. Then, we used two different ways, “PhyloCSF” and “ORF finder”, to find out whether CERS6-AS1 have the protein coding ability. LncRNA CERS6-AS1 is located on chromosome 2 and has 19 potential open reading frames (ORFs), however, there are no right ORFs by BLAST sequences (Supplementary Fig. 1A, B). Meanwhile, all the value of PhyloCSF were less than 0, indicating that CERS6-AS1 do not have protein coding ability by PhyloCSF track (Supplementary Fig. 1C). Summarily, the non-coding nature of CERS6-AS1 was confirmed.

**Fig. 1:**
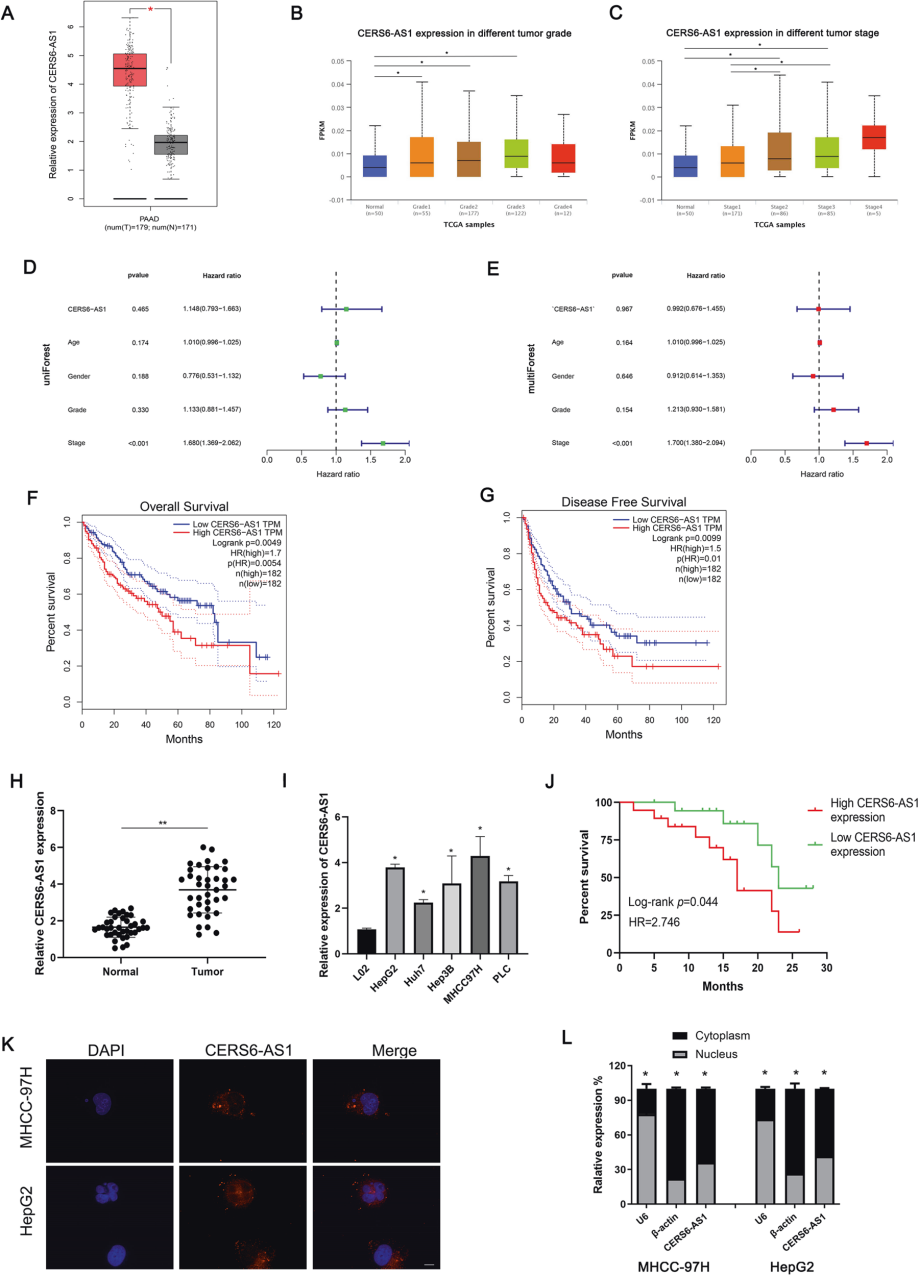
LncRNA CERS6-AS1 is high expression in HCC tissues and cell lines. **A** The expression levels of CERS6-AS1 between tumors and normal tissues based on GEPIA database. **B**, **C** The expression levels of CERS6-AS1 among different tumor Grade or Stage based on TCGA database. (Normal group is the control group). **D**, **E** Cox regression analysis based on TCGA database was performed to evaluate whether CERS6-AS1 is an independent risk factor of HCC prognosis, involving in age, gender, grade and stage. **F**, **G** Kaplan–Meier survival results of overall survival and disease-free survival based on CERS6-AS1 levels in HCC samples. **H** The expression of CERS6-AS1 in 38 paired HCC tissues and normal tissues. **I** CERS6-AS1 expression in HCC cell lines, LO2 cell line is the control group. **J** Kaplan–Meier survival results of overall survival based on CERS6-AS1 expression in HCC patients. (high CERS6-AS1 expression group, *n* = 19; low CERS6-AS1 expression group, *n* = 19). **K** FISH assay was performed to detect the location of CERS6-AS1. Scale bar: 50 μm. **L** Relative CERS6-AS1 expression in nucleus and cytoplasm. All experiments included were repeated at least three times, *p < 0.05, **p < 0.01.

Additionally, our team examined the RNA level of CERS6-AS1 in HCC data from Gene Expression Profiling Interactive Analysis (GEPIA) database which were based on The Cancer Genome Atlas (TCGA). We figured out that CERS6-AS1 was elevated in HCC tissues compared with adjacent normal tissues (Fig. 1A). Notably, CERS6-AS1 expression in grade 1 to 3 tumors was elevated compared with expression in normal tissues, whereas CERS6-AS1 expression in grade 4 tumors showed no significant difference compared with expression in normal tissues (Fig. 1B and Supplementary Fig. 2A). CERS6-AS1 expression in stage 2–3 HCC tissues was higher than that in normal tissues and stage 1 HCC tissues (Fig. 1C and Supplementary Fig. 2B). Subsequently, to clarify whether CERS6-AS1 was an independent risk factor of HCC prognosis, we utilized Cox regression analysis based on confounding factors, including age, gender, tumor stage. The results suggested that CERS6-AS1 was an independent risk factor of HCC stage by performing univariate and multivariate regression analysis (Fig. 1D, E).

We next examined survival data from GEPIA. Overall survival analysis indicated that high CERS6-AS1 expression level was significantly correlated with dismal prognosis (Fig. 1F), and similar results were obtained regarding disease-free survival (Fig. 1G).

We next investigated CERS6-AS1 expression in 38 fresh HCC tumor specimens and paired adjacent normal specimens using qRT-PCR analysis. The results illustrated that CERS6-AS1 was upregulated in all tumor specimens compared with paired normal specimens (Fig. 1H). Expression of CERS6-AS1 was also upregulated in five HCC cell lines, including HepG2, Huh7, Hep3B, MHCC97H and PLC, compared with the L02 cell line (Fig. 1I). CERS6-AS1 expression was highest in HepG2 and MHCC97H cell lines, and thus HepG2 and MHCC97H were selected for subsequent experiments. With our clinical data, overall survival also suggested that HCC patients with high CERS6-AS1 expression had a poor prognosis (Fig. 1J).

To determine the subcellular location of CERS6-AS1 in HCC cells, FISH assay was performed in HepG2 and MHCC97H cells. CERS6-AS1 was observed throughout the cell but it was mainly located in the cytoplasm (Fig. 1K). We performed qRT-PCR analysis in nuclear and cytoplasmic fractions of cells and the results also indicated CERS6-AS1 was mainly located in the cytoplasm (Fig. 1L). Together, these findings suggest that CERS6-AS1 may exert oncogenic functions for HCC progression.

### LncRNA CERS6-AS1 promotes HCC cell growth and glycolysis

**Fig. 2:**
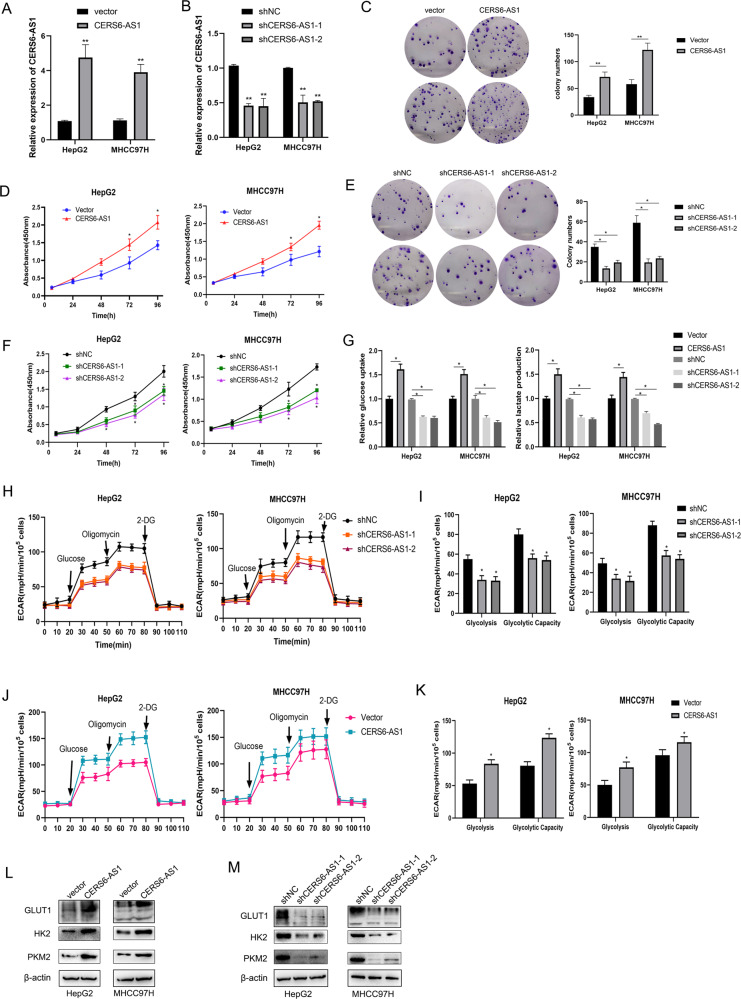
LncRNA CERS6-AS1 facilitates HCC cell proliferation and aerobic glycolysis. **A**, **B** The qRT-PCR analysis was utilized to measure the transfection efficiency in HepG2 and MHCC97H with CERS6-AS1 overexpression or knockdown sequence. Colony formation assays (**C**, **E**) and CCK-8 assays (**D**, **F**) were used to evaluate the proliferated ability in CERS6-AS1 overexpression or knockdown groups, respectively. **G** The relative glucose uptake or lactate production in CERS6-AS1 overexpression or knockdown groups. **H**, **J** ECAR of HepG2 or MHCC97H cells in CERS6-AS1 overexpression and knockdown groups were measured by a Seahorse Bioscience XFp analyzer. 2-DG 2-deoxy-d-glucose. **I**, **K** The statistical chart based on ECAR curve. **L**, **M** The key glycolysis proteins were detected in CERS6-AS1 overexpression or knockdown groups via western blot. All experiments included were repeated at least three times, *p < 0.05, **p < 0.01.

To examine the potential effect of CERS6-AS1 in HCC cells, we used lentivirus containing CERS6-AS1 overexpression or knockdown sequences to infect HepG2 and MHCC97H, respectively. Seven days after infection, we utilized qRT-PCR analysis to measure the RNA level of CERS6-AS1 and we confirmed successful modulation of CERS6-AS1 levels in HepG2 and MHCC97H cell lines by lentivirus vectors (Fig. 2A, B). Subsequently, we utilized these stable expression cell lines (about 10 days after infection) to evaluate the biological phenotype. The role of CERS6-AS1 in the proliferation of hepatocarcinoma cells in vitro was investigated by CCK-8 assay and colony formation assay. Upregulation level of CERS6-AS1 increased HCC cell growth, however knockdown of CERS6-AS1 significantly inhibited the proliferation ability (Fig. 2C–F).

Glucose metabolic reprogramming is essential for HCC cell proliferation. Thus, we next performed glucose uptake, lactate production and ECAR assays to identify the function of CERS6-AS1 on aerobic glycolysis in HCC cells. The results indicated that upregulation of CERS6-AS1 enhanced the glucose uptake and lactate production, whereas knockdown of CERS6-AS1 inhibited them (Fig. 2G). ECAR reflects the overall glycolytic flux, and a higher ECAR reflects increased glycolysis. Knockdown of CERS6-AS1 expression significantly inhibited the ECAR (Fig. 2H, J). On the basis of the ECAR results, we confirmed that silenced CERS6-AS1 expression markedly reduced the glycolysis and glycolytic capacity of HCC cells (Fig. 2I). However, CERS6-AS1 overexpression showed the reversed results (Fig. 2K).

We next evaluated the protein levels of GLUT1, HK2 and PKM2, which are essential proteins in the glycolysis signaling pathway. The results confirmed that CERS6-AS1 overexpression upregulated the protein levels of these proteins, whereas CERS6-AS1 knockdown reduced expression of the proteins (Fig. 2L, M and Supplementary Fig. 3A, B).

### LncRNA CERS6-AS1 facilitates HCC cell migration and invasion and induces epithelial-mesenchymal transition (EMT)

**Fig. 3:**
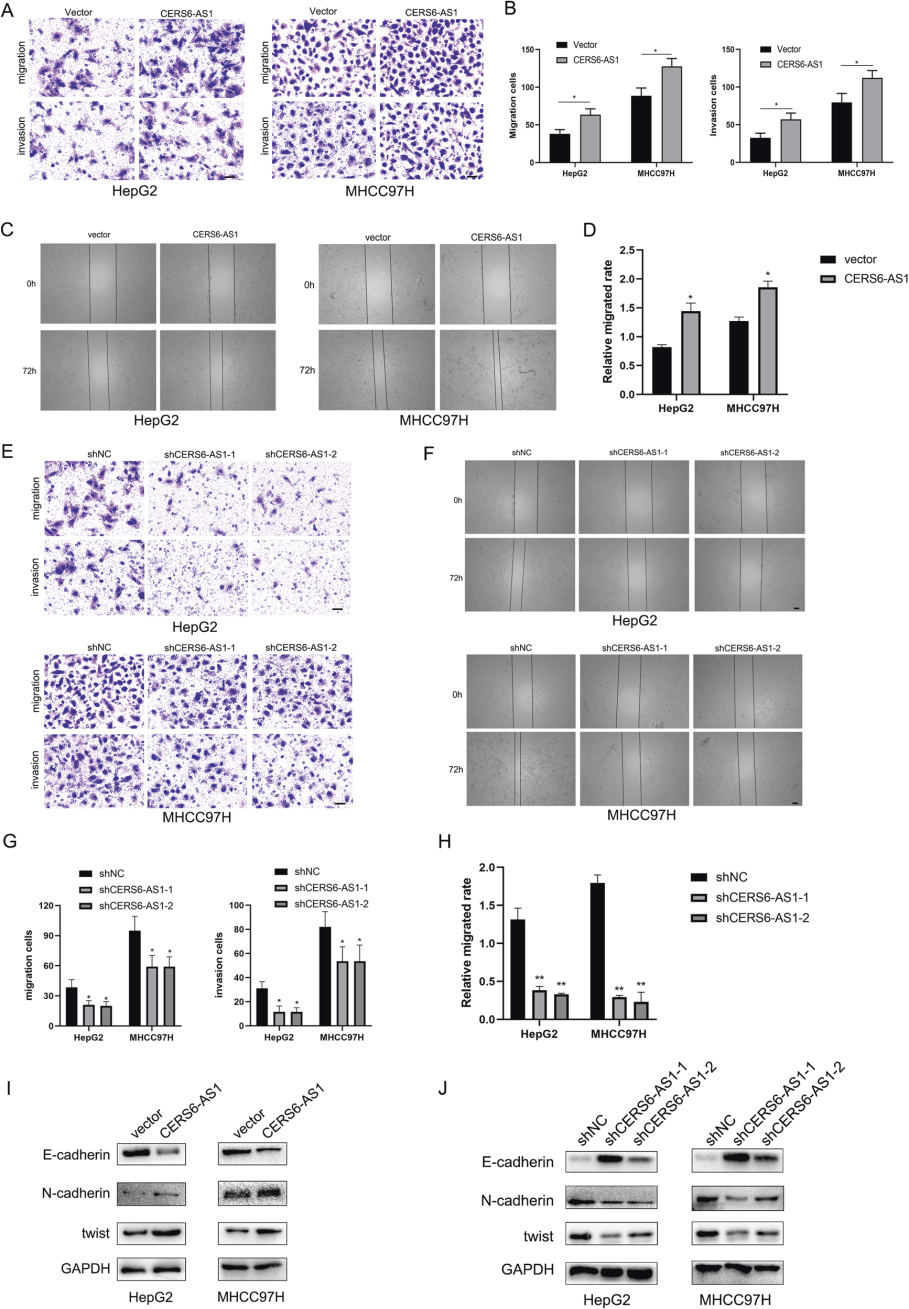
LncRNA CERS6-AS1 enhances HCC migration and invasion. **A**–**D** Transwell assay and wound healing assay were performed to evaluate the migration and invasion in CERS6-AS1 overexpression group. Transwell assay, Scale bar: 100 μm; Wound healing assay, Scale bar: 250 μm. **E**–**H** Transwell assay and Wound healing assay were performed to evaluate the migration and invasion in CERS6-AS1 knockdown groups. Transwell assay, Scale bar: 100 μm; Wound healing assay, Scale bar: 250 μm. **I**, **J** The protein markers of EMT, including E-cadherin, N-cadherin and Twist, were measured via western blot in CERS6-AS1 overexpression and knockdown groups. All experiments included were repeated at least three times, *p < 0.05, **p < 0.01.

Intra-hepatic tissue invasion and distant metastasis are vital malignant biological phenotypes of HCC. Next, transwell and wound healing experiments were performed to evaluate the effect of CERS6-AS1 on the migration and invasion ability of HCC cells. As displayed in Fig. 3A, B, CERS6-AS1 overexpression significantly facilitated the migration and invasion abilities of HCC cells in Transwell assay. The wound healing assays were consistent with these results (Fig. 3C, D). In contrast, CERS6-AS1 knockdown limited the migration and invasion abilities of HCC cells (Fig. 3E–H).

Research has established the important role of EMT in tumor cell migration. Western blot analysis of key markers of EMT showed that overexpression of CERS6-AS1 upregulated the protein expressions of N-cadherin and twist and inhibited E-cadherin expression (Fig. 3I and Supplementary Fig. 3C). Knockdown of CERS6-AS1 showed the opposite results (Fig. 3J and Supplementary Fig. 3D).

Together, these results suggest that CERS6-AS1 facilitates HCC cell migration and invasion and induces EMT.

### LncRNA CERS6-AS1 sponges miR-30b-3p and abolishes the inhibition of miR-30b-3p on its target gene MDM2

**Fig. 4:**
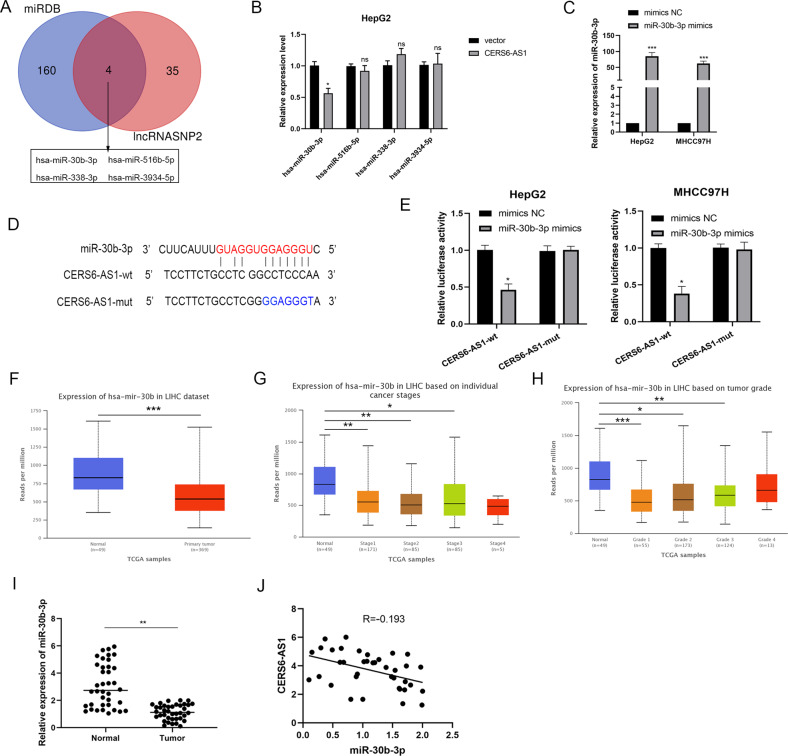
LncRNA CERS6-AS1 sponges to miR-30b-3p. **A** Venn analysis showed the potential target miRNA, including miR-30b-3p, miR-516b-5p, miR-338-3p, miR-3934-5p. **B** The relative expression of these miRNAs after transfecting CERS6-AS1 overexpression sequence or empty sequence. **C** The transfection efficiency of miR-30b-3p mimics in HCC cells were measured by qRT-PCR analysis. **D** The binding site between miR-30b-3p and CERS6-AS1. **E** luciferase assay was performed to detect the direct interaction between CERS6-AS1 and miR-30b-3p. **F**–**H** The expression of miR-30b-3p in HCC tumors and normal tissues based on individual cancer stage or grade from TCGA database, normal group regards as control group. **I** The expression of miR-30b-3p in 38 paired HCC tissues were measured by PCR analysis. **J** Pearson correlation analysis was performed to evaluate the correlation between miR-30b-3p and CERS6-AS1. All experiments included were repeated at least three times, *p < 0.05, **p < 0.01, ***p < 0.001.

Our results indicated that CERS6-AS1 is mainly located in the cytoplasm of HCC cells, suggesting that CERS6-AS1 can exert as a ceRNA. To explore the mechanism of CERS6-AS1 in HCC, we screened potential miRNA targets using two different bioinformatics prediction networks. We identified 164 potential miRNAs in miRDB (http://mirdb.org/) and 39 potential miRNAs in lncRNASNP2 (http://bioinfo.life.hust.edu.cn/lncRNASNP2). Venn diagram analysis identified four overlapping miRNAs: miR-30b-3p, miR-516b-5p, miR-338-3p and miR-3934-5p (Fig. 4A). We next evaluated miRNA expressions in HCC cells with CERS6-AS1 overexpression using qRT-PCR analysis. PCR analysis demonstrated that only miR-30b-3p was decreased in response to CERS6-AS1 overexpression; the other miRNAs showed no changes (Fig. 4B).

To more closely examine the regulatory relationship and interaction between miR-30b-3p and CERS6-AS1, we performed luciferase reporter assays. We upregulated miR-30b-3p by transfecting cells with miR-30b-3p mimics and confirmed induced expression by qRT-PCR analysis (Fig. 4C). The results illustrated that CERS6-AS1 had the interacted site between CERS6-AS1 and miR-30b-3p and we constructed the wild-type and mutant-type plasmids (Fig. 4D). Luciferase reporter assay showed that CERS6-AS1 expression in wild-type decreased by upregulating miR-30b-3p expression, whereas CERS6-AS1 in mutant-type had no significant (Fig. 4E).

We next analyzed miR-30b-3p expression level through TCGA database, and found that the RNA level of it was remarkably low in HCC tumor tissues compared to normal tissues (Fig. 4F and Supplementary Fig. 2C). Subsequently, miR-30b-3p expression in stage 1–3 and grade 1–3 tissues was lower than that in normal tissues (Fig. 4G, H and Supplementary Fig. 2D, E). Meanwhile, we evaluated miR-30b-3p expression in our paired tumor samples, revealing that miR-30b-3p was dramatically decreasing in HCC samples compared to normal samples (Fig. 4I). Subsequently, Pearson correlation analysis illustrated that CERS6-AS1 was negatively associated with miR-30b-3p (Fig. 4J).

**Fig. 5:**
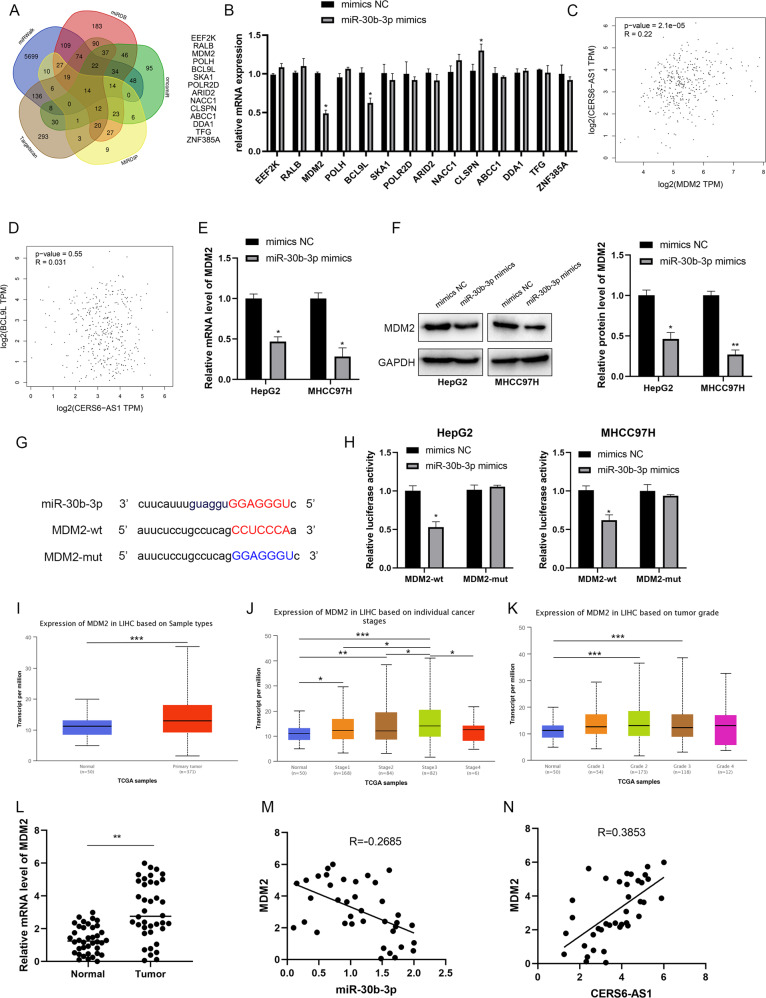
LncRNA CERS6-AS1 upregulates MDM2 via sponging to miR-30b-3p. **A** Venn analysis screened 14 potential target genes of miR-30b-3p. **B** The mRNA expression levels of these potential target genes was measured by PCR analysis. The correlation between CERS6-AS1 and MDM2 (**C**) or BCL9L (**D**) was evaluated based on TCGA database. **E**, **F** The relative mRNA and protein expression of MDM2 in miR-30b-3p mimics group and negative group. **G** The binding site between miR-30b-3p and MDM2. **H** Luciferase assay was performed to detect the direct interaction between MDM2 and miR-30b-3p. **I-K** The expression of MDM2 in HCC tumors and normal tissues based on individual cancer stage or grade from TCGA database, normal tissues regard as control group. **L** The expression of MDM2 in HCC tissues was measured by PCR analysis. **M** Pearson correlation analysis was performed to evaluate the correlation between miR-30b-3p and MDM2. **N** The correlation between MDM2 and CERS6-AS1 was evaluated via Pearson correlation analysis. All experiments included were repeated at least three times, *p < 0.05, **p < 0.01, ***p < 0.001.

We next used five bioinformatics prediction networks (miRDB, miRDIP, oncomiR, Targetscan and miRWalk) to screen candidate genes and 14 potential target genes were selected (Fig. 5A). PCR analysis was utilized to evaluate the mRNA levels of target genes when miR-30b-3p mimics were successful transfected into HepG2 cells. The results confirmed that MDM2 and BCL9L were downregulated after miR-30b-3p upregulation (Fig. 5B). Based on TCGA database, Pearson correlation analysis revealed MDM2, an E3 ubiquitin ligase involved in ubiquitin-mediated proteolysis with critical functions in cancer, was positively correlated with CERS6-AS1 and chosen among the candidate genes for further study (Fig. 5C). However, another candidate gene, BCL9L, was not significantly associated with CERS6-AS1 expression (Fig. 5D).

Subsequently, we respectively evaluated the expression levels of MDM2 mRNA and protein in cells transfected with miR-30b-3p mimic. Interestingly, it suggested that miR-30b-3p overexpression significantly suppressed MDM2 expression levels (Fig. 5E, F). Luciferase reporter assay confirmed that MDM2 is negatively regulated by miR-30b-3p (Fig. 5G, H). Analysis of TCGA indicated that MDM2 is highly expressed in HCC samples. Meanwhile, tumors with higher grade or stage showed higher expressions of MDM2 (Fig. 5I–K and Supplementary Fig. 2F–H). Meanwhile, we found MDM2 was upregulated in HCC tissues compared to their paired adjacent tissues (Fig. 5L). Pearson correlation analysis suggested that MDM2 was negatively associated with miR-30b-3p, while positively correlated with CERS6-AS1 (Fig. 5M, N).

### LncRNA CERS6-AS1 promotes HCC proliferation, migration, invasion and glycolysis via sponging miR-30b-3p

**Fig. 6:**
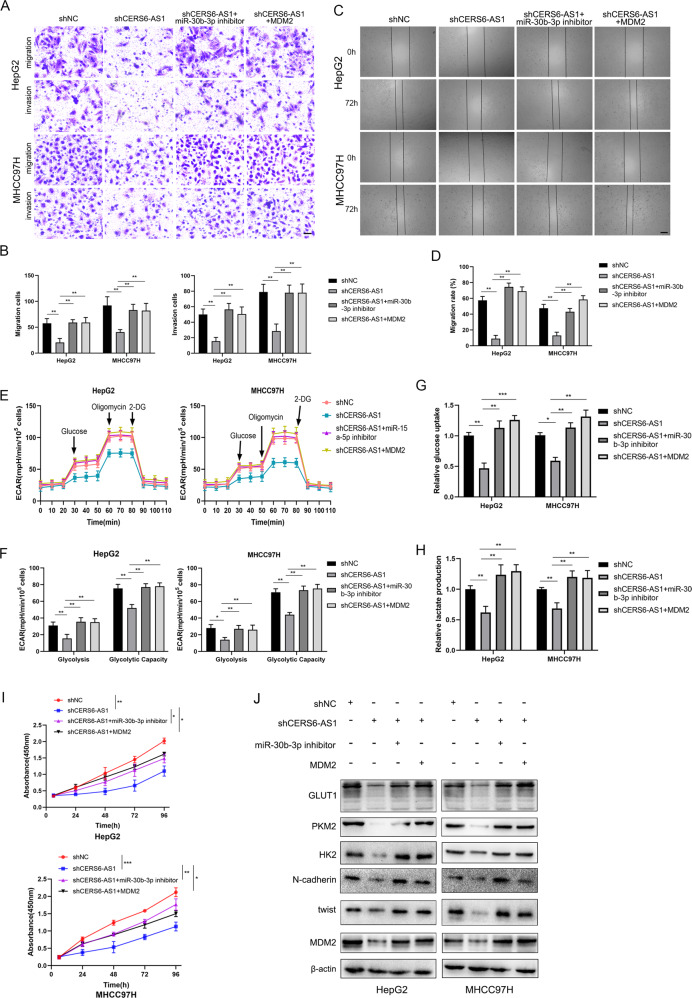
LncRNA CERS6-AS1 facilitates HCC progression via modulating MDM2 expression. **A**–**D** MDM2 overexpression or miR-30b-3p inhibitors partly reversed the inhibition effect of CERS6-AS1 knockdown on migration and invasion of HepG2 and MHCC97H. Transwell assay, Scale bar: 100 μm; Wound healing assay, Scale bar: 250 μm. **E**–**H** MDM2 overexpression or miR-30b-3p inhibitors partly reversed the inhibition effect of CERS6-AS1 knockdown on aerobic glycolysis of HepG2 and MHCC97H. **I** MDM2 overexpression or miR-30b-3p inhibitors partly reversed the inhibition effect of CERS6-AS1 knockdown on proliferation of HepG2 and MHCC97H. **J** MDM2 overexpression or miR-30b-3p inhibitors partly reversed the inhibition effect of CERS6-AS1 knockdown on protein expression involved in EMT (N-cadherin, twist) and glycolysis (PKM2, GLUT1). *p < 0.05, **p < 0.01.

To prove the carcinogenic effect of CERS6-AS1 caused by the adsorption of target gene miR-30b-3p, we conducted a series of functional rescue experiments. Functional rescue experiments suggested that knockdown CERS6-AS1 expression plus miR-30b-3p inhibitor or MDM2 could partially abolish the inhibition of knockdown CERS6-AS1 on migration, invasion, proliferation and glycolysis (Fig. 6A–I). Western blot analysis showed that CERS6-AS1 knockdown–mediated inhibitory effects on GLUT1, PKM2, HK2, N-cadherin and twist protein levels were abrogated by miR-30b-3p inhibitors or MDM2 (Fig. 6J and Supplementary Fig. 3E).

### LncRNA CERS6-AS1 promotes MDM2-mediated ubiquitination of p53

**Fig. 7:**
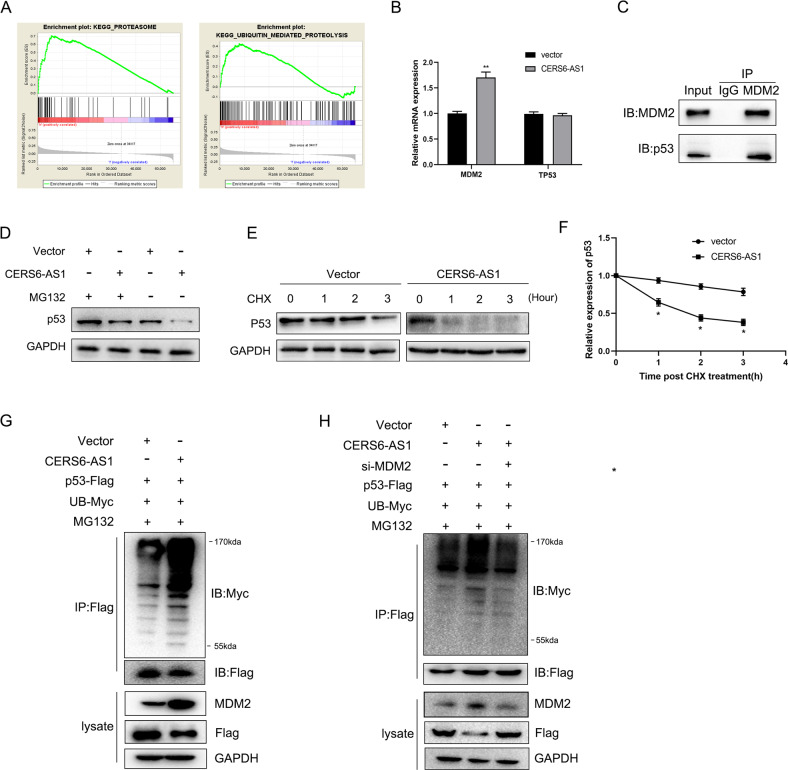
LncRNA CERS6-AS1 upregulates MDM2 and promotes MDM2-mediated ubiquitin-dependent degradation of p53. **A** GSEA analysis indicated that CERS6-AS1 was significantly correlated with proteasome and ubiquitin-mediated proteolysis. **B** qRT-PCR analysis was performed to evaluate the expression of MDM2 and TP53 in CERS6-AS1 overexpression group. **C** The interaction between MDM2 and p53 was verified by co-immunoprecipitation assay. **D** The protein stability of p53 were evaluated in Vector group and CERS6-AS1 overexpression group with the treatment of MG132 (4 h, 5 μM) in HepG2. **E** The protein levels change of p53 in CERS6-AS1 overexpression and control groups when HepG2 cells were treated with cycloheximide (CHX, 50 μg/ml) for 0–3 h, respectively. **F** The curve of CHX treatment showed the change of MDM2 protein expression. **G** The ubiquitination levels of p53 in CERS6-AS1 overexpression and control groups. **H** Silence MDM2 partly reverse the promoting effect of CERS6-AS1 overexpression on p53 ubiquitination.

Through GSEA analysis, we found that CERS6-AS1 was positively correlated with proteasome and ubiquitin-mediated proteolysis (Fig. 7A). Thus, we hypothesized that the effects of CERS6-AS1 in HCC cells may involve ubiquitination. MDM2 interacts with and negatively regulates the p53 tumor suppressor by promoting its ubiquitin-dependent proteasomal-mediated degradation [15, 16]. Therefore, we measured the mRNA levels of MDM2 and p53 in CERS6-AS1 overexpressing cells and found that CERS6-AS1 overexpression upregulated the mRNA levels of MDM2 but had no influence on p53 mRNA levels (Fig. 7B). Immunoprecipitation assay suggested that there was an interaction between MDM2 and p53 (Fig. 7C). Subsequently, MG132, a proteasome inhibitor, was performed to evaluate whether MG132 affected p53 protein stability. The results illustrated that the protein levels of p53 was decreased in HepG2 cells with CERS6-AS1 overexpression, while MG132 could inhibit the degradation of p53 and enhanced p53 protein stability (Fig. 7D and Supplementary Fig. 3F). Meanwhile, protein stability experiments using cycloheximide revealed that CERS6-AS1 overexpression significantly accelerated the degradation of p53 protein (Fig. 7E, F).

We next examined the effect of CERS6-AS1 on the ubiquitination of p53. We transfected Flag-tagged p53 and Myc-labeled ubiquitin plasmids into CERS6-AS1-overexpressing HepG2 cells. The MG132 proteasome inhibitor was added 4 h before protein extraction. The results elucidated that CERS6-AS1 overexpression significantly accelerated the ubiquitination of p53 (Fig. 7G). However, when cells were co-transfected with MDM2 shRNA along with CERS6-AS1 overexpression constructs, the ubiquitination level was suppressed (Fig. 7H). Together, these results suggest that CERS6-AS1 promoted the ubiquitin-dependent proteasomal degradation of p53 by upregulating the E3 ligase MDM2.

### LncRNA CERS6-AS1 promote HCC tumor growth in vivo

**Fig. 8:**
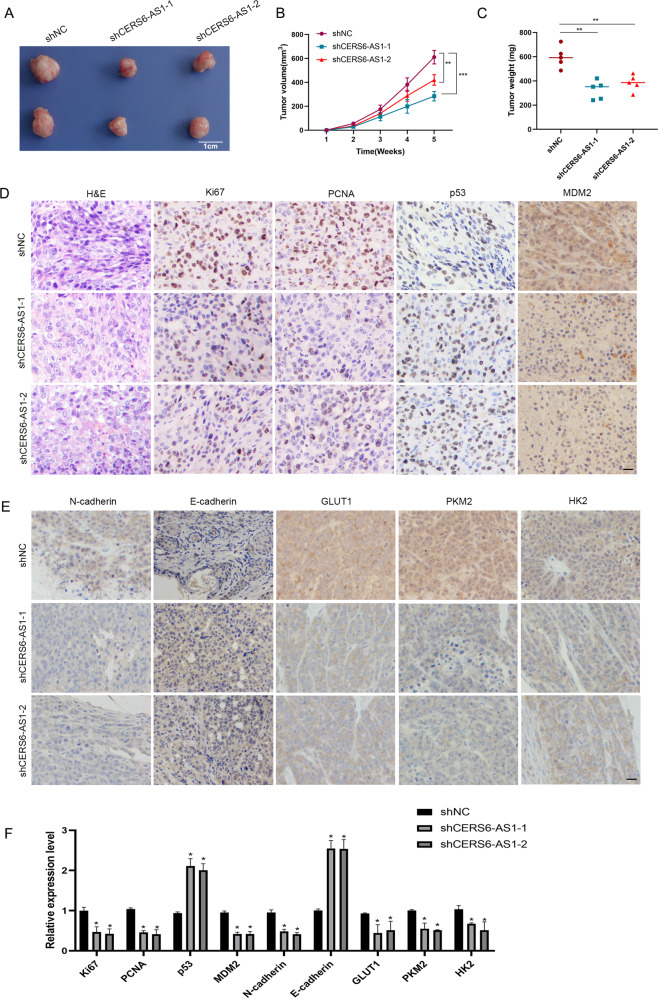
LncRNA CERS6-AS1 accelerates HCC proliferation in vivo. **A** Morphologic characteristics of xenograft tumors from HepG2/shNC group and HepG2/shCERS6-AS1 group (*n* = 5, scale bar: 1 cm). **B** The volume of xenograft tumors. **C** The weight of xenograft tumors. **D** Representative images of H&E, Ki67, PCNA, p53 and MDM2 staining in the xenograft tumors and the relative expression levels in shNC and shCERS6-AS1 groups. Scale bar: 100 μm. **E** Representative images of MDM2, N-cadherin, E-cadherin, GLUT1, PKM2 and HK2 staining in the xenograft tumors and the relative expression levels in shNC and shCERS6-AS1 groups. Scale bar: 100 μm. **F** The statistical analysis of these protein expression levels in shNC and shCERS6-AS1 groups. *p < 0.05, **p < 0.01.

To explore the role of lncRNA CERS6-AS1 in HCC in vivo, we constructed a subcutaneous tumorigenesis model by injecting nude mice with HCC cells expressing CERS6-AS1 knockdown constructs or controls. The results showed that CERS6-AS1 knockdown remarkably suppressed tumor growth (Fig. 8A). Knockdown of CERS6-AS1 expression significantly reduced the size of tumors compared with the negative control group (Fig. 8B). The tumor weight was also smaller in the CERS6-AS1 knockdown group (Fig. 8C). H&E staining illustrated CERS6-AS1 led to increased cell malignancy, including large nuclei, abnormal division, hyperchromatic, and obvious atypia (Fig. 8D). Ki67 and PCNA proliferation indicators were downregulated in tumors from the CERS6-AS1 knockdown groups as detected by immunohistochemical (IHC) assays (Fig. 8D). We also evaluated p53, MDM2, N-cadherin, E-cadherin, GLUT1, HK2 and PKM2 expression in tumors. The results revealed decreased MDM2, N-cadherin, GLUT1, HK2 and PKM2 expression in the CERS6-AS1 knockdown tumors, whereas E-cadherin and p53 were highly expressed (Fig. 8D–F). Together these results demonstrated that CERS6-AS1 promotes HCC tumor growth in vivo.

## Discussion

Multiple studies have indicated that CERS6-AS1 may regulate tumor progression and influence disease outcome. Bao et al. reported that CERS6-AS1 directly binds to IGF2BP3, a RNA-binding protein, and enhances the stability of CERS6 mRNA, thereby promoting the progression of breast tumors [17]. Yan et al. uncovered that CERS6-AS1 can facilitate breast cancer cell growth and limit cell apoptosis via binding to miR-125a-5p to block the miR-125a-5p-mediated inhibitory function on BAP1 expression [18]. Xu et al. also found that CERS6-AS1 served as molecular sponges to bind miRNA and promote downstream gene expression in pancreatic cancer. CERS6-AS1 competitively binds miR-217 to indirectly regulate the expression of YWHAG, which interacts with RAF1 and activates the ERK signaling, thereby accelerating pancreatic cancer progression [19]. In another report of pancreatic cancer, CERS6-AS1 sequesters miR-15a-5p, which induces the expression of FGFR1 and HMGA1, two key oncogenes, promoting pancreatic cancer proliferation and migration [20, 21]. Studies in non-tumor diseases have indicated that CERS6-AS1 might serve crucial functions in Alzheimer’s disease and right ventricular cardiomyopathy induced by tricuspid regurgitation [22, 23].

In our report, our team explored the malignant phenotype and molecular mechanism of lncRNA CERS6-AS1 in HCC samples and cells. Results uncovered that the RNA level of lncRNA CERS6-AS1 was both remarkably elevated in HCC samples and cancer cells, which was consistent with the study by Zhang et al. The authors used a lncRNA network analysis linked to the diagnosis and prognosis of HCC and also suggested that CERS6-AS1 was high expression and its upregulation indicated poor prognosis [24]. However, the previous study did not examine the function and mechanism in HCC. Here we expanded these findings by performing in vitro and in vivo experiments to elucidate the role of CERS6-AS1 in HCC progression.

Date from TCGA database indicated that the level of lncRNA CERS6-AS1 was increased in HCC tissues and its higher expression was more correlated with dismal prognosis. We further confirmed CERS6-AS1 was upregulated in HCC tissues and cell lines. Meanwhile, it promoted HCC cell growth and metastasis and enhanced aerobic glycolysis. Our results indicate that CERS6-AS1 binds miR-30b-3p to reverse the inhibitory effect of miR-30b-3p on MDM2 expression. Rescue experiments showed that CERS6-AS1 facilitated HCC cell growth, invasion and glycolytic remodeling by exerting as a sponge for miR-30b-3p to indirectly increase MDM2 expression. Therefore, this evidence confirmed that CERS6-AS1 enhanced HCC malignant phenotypes and glycolysis by sponging miR-30b-3p to increase MDM2 expression, thereby facilitating p53 ubiquitination and weakening its tumor suppressive effect on HCC progression.

MDM2 is E3 ubiquitin ligase that participates in ubiquitin-dependent proteasome degradation of target proteins. In previous study, Haupt et al. proved that the rapid degradation of p53 was mediated by MDM2 [25]. The protein stability and function of p53 are regulated by MDM2, which directly inhibits p53 transcriptional activity by binding to its transcriptional activation domain and targets p53 for degradation [26]. In order to maintain the long-term survival of hepatocytes, hepatitis virus induces TP53 mutation and silencing, promotes MDM2 overexpression, which ultimatedly accelerates p53 degradation and stabilizes MDM2 [27]. Thus, induced genetic instability, endoplasmic reticulum stress, energy metabolism switches, oxidative stress and antitumor gene abnormalities may promote hepatocellular transformation into HCC cells [28, 29].

Glycolytic remodeling is a crucial hallmark of HCC involved in tumor progression and undesirable clinical outcomes [30]. The lncRNA SOX2OT enhanced HCC invasion and migration by sponging miR-122-5p and activating PKM2, which is a crucial enzyme in glycolysis progression [31]. The lncRNA KCNQ1OT1 competitively binds to miR-34c-5p to indirectly upregulate ALDOA and facilitates aerobic glycolysis, promoting osteosarcoma proliferation [32]. Interestingly, LINC01133 increases c-Myc expression by competitively binding to miR-199a-5p. Meanwhile, LINC01133 is a direct transcriptional target of c-Myc. This positive feedback promotes c-Myc-mediated transcriptional regulation of HK2 and LDHA, accelerating the aerobic glycolysis of lung cancer [33]. Intriguingly, this study demonstrated that CERS6-AS1 facilitated the expressions of GLUT1, PKM2 and HK2, which are key enzymes for glucose metabolism. CERS6-AS1 promoted MDM2-mediated ubiquitin-dependent degradation of p53, blocking the inhibitory effect of p53 on glucose metabolism. Previous studies showed that p53 inhibits glucose transporters GLUT1 and GLUT4 through transcriptional repression, and negatively regulates glycolysis by transactivation of RRAD and TIGAR, which are two key suppressors of glycolysis [34–36]. In addition, p53 can directly interact with and suppress glucose-6-phosphate dehydrogenase, thereby inhibiting glucose metabolism [37]. Thus, CERS6-AS1 involved in regulation of p53 might control glucose metabolism and HCC progression. However, this study also has deficiencies. For example, CERS6-AS1 may also act in cis and either via RNA, act of transcription or the chromatin status of the locus affect the expression of nearby genes which may regulate metabolism. Thus, we may discuss this aspect in the further study.

**Fig. 9:**
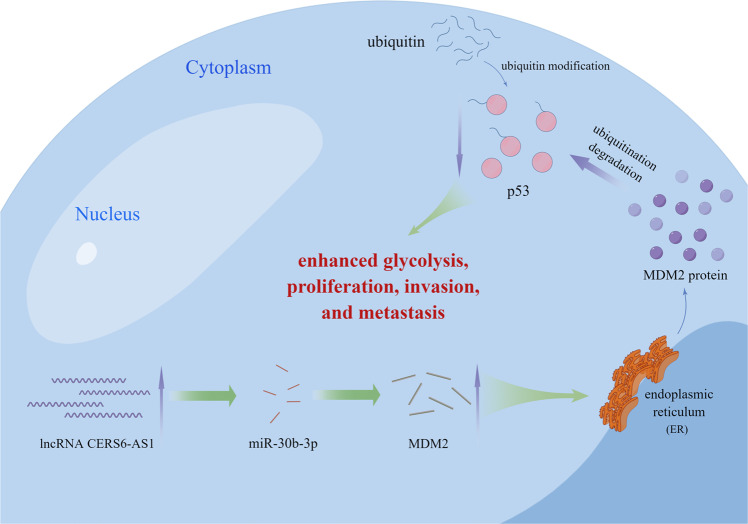
The mechanism diagram of CERS6-AS1 promoting HCC progression. CERS6-AS1 sponges miR-30b-3p and upregulates MDM2, leading to increase of p53 degradation by ubiquitination. Eventually, CERS6-AS1 promotes cell proliferation, migration and invasion and induces cellular metabolic remodeling. (The diagram is drawn by Figdraw (www.figdraw.com)).

In summary, we showed that CERS6-AS1 expression was overexpressed in HCC samples and cell lines. High expression of CERS6-AS1 was positively correlated with miserable prognosis of HCC patients. CERS6-AS1 promoted HCC cell growth and metastasis and accelerated aerobic glycolysis. Mechanistically, CERS6-AS1 elevated the expression of MDM2 by functioning as a sponge for miR-30b-3p, promoting MDM2-mediated ubiquitin-dependent degradation of p53 (Fig. 9). Our study suggests that the CERS6-AS1/miR-30b-3p/MDM2/p53 signaling axis may represent a novel regulation mechanism in HCC, and CERS6-AS1 may be new therapeutic target of HCC.

## Materials and methods

### Cell lines and clinical samples

The L02, HepG2, PLC, Huh7, Hep3B and MHCC97H cell lines were purchased from the American Type Culture Collection (USA). Among them, L02, HepG2, PLC, Hep3B and MHCC97H cells were cultured in Dulbecco’s Modified Eagle’s Medium (Gibco, NY, USA) containing 10% fetal bovine serum (FBS). Huh7 cells were cultured in RPMI-1640 medium supplemented with 10% FBS. All cell lines were cultured in a 37 °C incubator with 5% CO_2_.

Thirty-eight paired frozen HCC tissues and normal liver specimens were obtained from HCC patients who underwent radical hepatectomy in the West China Hospital of Sichuan University from 2019 to 2021. All patients gave informed consent and signed relevant consent forms. The Clinical Research Ethics Committees of the West China Hospital of Sichuan University approved this study.

### Quantitative RT-PCR

Total RNA was extracted from tissues and cells using RNA-easy Isolation Reagent (Vazyme, China). RNA was transcribed to cDNA using the HiScript III 1st Strand cDNA Synthesis Kit (+gDNA wiper) (Vazyme). The miRNA 1st Strand cDNA Synthesis Kit (by stem-loop) (Vazyme) was used for miRNA reverse transcription. Real-time PCR was performed with ChamQ Universal SYBR qPCR Master Mix (Vazyme) and miRNA Universal SYBR qPCR Master Mix (Vazyme). GAPDH mRNA, as an endogenous reference gene, was used to normalize target gene expression and U6 was used for normalizing miRNA. Gene expression was calculated using an optimized comparative Ct (2^ΔΔCt^) value method. All RNA primer sequences are shown in Table 1.

**Table 1:** The primer of genes involved in this study.

Gene symbol	Sequence (5′-3′)	
	Forward primer	Reverse primer
CERS6-AS1	AAGACCAGCCTGGCCAATAT	GCCTCCTGAGTAGCTGGAAT
MiR-30b-3p	TGCGGAGAGGTTGCCCTTGGTGA	TGCGGGTGCTCGCTTCGGCAGC
MDM2	CAGTAGCAGTGAATCTACAGGGA	CTGATCCAACCAATCACCTGAAT
TP53	GAGGTTGGCTCTGACTGTACC	TCCGTCCCAGTAGATTACCAC
GAPDH	CTGGGCTACACTGAGCACC	AAGTGGTCGTTGAGGGCAATG
U6	TGCGGGTGCTCGCTTCGGCAGC	CCAGTGCAGGGTCCGAGGT

### Stable knockdown and overexpression of CERS6-AS1 in HCC cells

Lentiviral vectors containing sequences for knockdown or overexpression of CERS6-AS1 were constructed by and purchased from Genechem (China) (Supplementary Table 1), and the vectors were used following the manufacturer’s recommendations. The amount of lentivirus was calculated according to MOI value and lentivirus titer value of cell lines. Lentivirus was added to cells (2 × 10^5^ cells). We collected virus particles 48 h after infection and used them to infect HCC cells to generate stable cell lines.

### LncRNA fluorescence in situ hybridization (FISH) assay

FISH assay was performed using the Cy3-labeled lncRNA CERS6-AS1 probe and the Ribo™ Fluorescent In Situ Hybridization Kit (RiboBio, China). The cells were fixed on a slide with 4% paraformaldehyde, and 0.5% Triton X-100 was used for cell membrane perforation. Cells were washed with PBS, and pre-hybridization buffer and hybridization buffer were added to cells in turn. DAPI was applied to stain nuclei, and anti-fluorescence quenching PVP sealing liquid (Boster, China) was added for sealing. Cells were analyzed by fluorescence microscopy and images were obtained for analyses.

### Cell proliferation assays

For colony formation assays, ~1000 stable transfected HCC cells were respectively seeded into a 6-well plate and cultured for 12 days. After that, cancer cells were fixed with 4% paraformaldehyde for 30 min, followed by staining with 0.1% crystal violet for another 30 min. The colonies were washed with PBS to remove crystal violet staining solution, and the stained colonies were subsequently photographed.

For the Cell Counting Kit-8 (CCK-8) assay, ~2000 stable transfected HCC cells were respectively seeded in 96-well plates and cultured for 4 days. Next, 10% CCK-8 reagent (Dojindo, Japan) was prepared and separately added to cells in each well to incubate at 37 °C. After incubating 1 h, absorbance was evaluated by a 450 nm microplate reader once a day for 4 days and then curves were drawn.

### Migration and invasion assays

For Transwell assays, stable transfected HCC cells (1 × 10^5^ cells/well) were respectively seeded in the upper chamber with 200 μl medium containing 5% FBS, and the lower chamber was filled with 800 μl medium containing 20% FBS. For invasion assays, Matrigel was diluted to 10% and separately added to the upper chamber (10% Matrigel 100 μl). The plates were incubated in an incubator for 24–48 h and next the cells in lower chamber were then fixed with 4% paraformaldehyde and stained with 0.1% crystal violet. The numbers of migrated cells and invaded cells were evaluated in three random visual fields under a microscope.

For wound healing assays, stable transfected HCC cells were respectively plated in a six-well plate and cultured until they reached 90% confluence. A sterile plastic blade was utilized to scratch the cellular layer, and floating cells were removed by washing with PBS. The remaining adherent cells continue to be given 1% FBS to culture for another 3 days. The wound width was recorded every day and cell migration was analyzed using Image J software.

### Glucose uptake, lactate production and extracellular acidification rate (ECAR) assays

The Extracellular Flux Analyzer XF96 (Seahorse Bioscience, Billerica, MA, USA) was used to measure cellular glycolysis capacity using Glycolysis Stress Test Kit (Seahorse Bioscience), according to the manufacturer’s instructions. Cells (4 × 10^4^ cells/well) were seeded in XF96 cell culture microplates. For ECAR assays, 10 mM glucose, 1 μM oligomycin and 100 mM 2-deoxy-glucose (2-DG) were added to evaluate ECAR values. The values of ECAR was normalized based on the numbers of cells and the curve was drawn. For glucose uptake and lactate production, cells were separately cultured in 6-well plate and incubated for 24 h, subsequently, cell supernatant was collected to calculate the glucose concentration and lactate level by using Glucose Colorimetric assay kit (BioVision, USA) and Lactate Colorimetric Assay Kit II (BioVision, USA), respectively. The data were normalized to total cell number of each sample.

### Luciferase reporter assay

The wild-type fragment of CERS6-AS1 or MDM2 containing putative miR-30b-3p binding site and the mutant fragment of CERS6-AS1 or MDM2 were synthesized and cloned into the psiCHECK-2 luciferase reporter (Promega, Madison, WI, USA). By using Lipofectamine 3000, the negative control or overexpression of miR-30b-3p was co-transfected in HepG2 and MHCC97H cells together with the appropriate luciferase reporter construct. After 48 h, Renilla and firefly luciferase activities were measured via a Dual-luciferase Assay System (Promega, USA).

### Western blot

The cells were lysed by using RIPA buffer containing protease and phosphatase inhibitor cocktails (Boster). Samples were centrifuged 15,000 × *g* for 0.5 h at 4 °C, and then loading buffer was respectively added into the supernatant, followed by heating for 10 min at 95 °C. Protein samples were separated by SDS-PAGE and transferred to PVDF membranes. Membranes were blocked with 5% defatted milk for 2 h and then incubated with primary antibodies against anti-GLUT1 (21829-1-AP), anti-HK2 (22029-1-AP), anti-PKM2 (15822-1-AP), anti-E-cadherin (20874-1-AP), anti-N-cadherin (22018-1-AP), anti-twist (11752-1-AP), anti-GAPDH (10494-1-AP), anti-p53 (10442-1-AP), and anti-MDM2 (66511-1-Ig) (all 1:500) overnight at 4 °C. All primary antibodies were purchased from Proteintech (China). The membranes were washed with TBST and incubated with secondary HRP-conjugated antibody for 2 h at 37 °C. Protein bands were visualized with ECL Chemiluminescence substrate (Abclonal).

### In vivo tumor growth assay

Female nude mice (BALB/c-nu, 6 weeks old) were purchased from Beijing Weitong Lihua Laboratory Animal Technology Co., LTD (China). HepG2 cells were infected with lentivirus containing CERS6-AS1 knockdown or negative control sequences. Each experimental group were randomly divided into five mice. Subsequently, nude mice were subcutaneously injected with PBS suspension cells (1 × 10^6^), and fed and drank freely during the 12 h light /12 h dark cycle. Tumor volume and mice weight were measured weekly. At 5 weeks after inoculation, mice were killed. The tumors were removed, fixed with 4% paraformaldehyde and subjected to IHC analysis. The animal experiments were approved by the animal ethical review committee from West China Hospital of Sichuan University.

### IHC staining

A standard streptavidin-biotin-peroxidase complex method were utilized to perform IHC staining. In simple terms, the xenograft tumor tissues were paraffin-embedded and cut into 3-μm sections. After a series of steps including dewaxing, rehydrating, rinsing, blocking and antigen retrieval, the sections were incubated with primary antibodies at 4 °C overnight. The following antibodies were used: anti-Ki67, anti-PCNA, anti-p53, anti-MDM2, anti-N-cadherin, anti-E-cadherin, anti-GLUT1, anti-PKM2 and anti-HK2 (All 1:200). Subsequently, the slides were incubated with the Envision detection system (DAKO) and the nuclei were restained with Meyer hematoxylin. The pictures were captured under a microscope and the results were scored according to the percentage of positive-staining cells.

### Statistical analysis

In this study, all experiments included were repeated at least three times. All statistical data were calculated via SPSS 20.0 software and GraphPad Prism 8 software. The data are expressed as mean ± standard deviation. Two-tailed Student’s *t* test or one-way ANOVA was used to calculate the statistical significance between different groups. The survival rates in two groups were calculated using the log-rank test and Kaplan–Meier method. All results were confirmed from triplicate independent experiments. *p* < 0.05 indicated statistical significance.

## Supplementary information

Ssupplementary Figure and table legends

Supplemental Table 1

supplemental Figure 1

supplemental Figure 2

supplemental Figure 3

Original Data File

English editing Certificate

## Data Availability

All data generated and analyzed during this study are included in this published article are available on request.
