# Supplementary material for: Long noncoding RNA CERS6-AS1 modulates glucose metabolism and tumor progression in hepatocellular carcinoma by promoting the MDM2/p53 signaling pathway
Source: Cell Death Discov. 2022 Aug 4;8:348. doi: 10.1038/s41420-022-01150-x (PMC9352870; doi:10.1038/s41420-022-01150-x)
Supplement: Supplementary file 1 — Ssupplementary Figure and table legends [file 41420_2022_1150_MOESM1_ESM.docx]

**Supplementary figure and table legends**

**Supplementary Table 1. The sequences of shRNA in this study.**

**Supplementary Figure 1. lncRNA CERS6-AS1 dose not have the ability to encode proteins.** (A) The potential open reading frame (ORF) in CERS6-AS1 was analyzed by using ORF finder via the sequences of CERS6-AS1(https://www.ncbi.nlm.nih.gov/orffinder/). The red zones represent the position of the ORF in the sequence. (B) The detail of ORF displayed in the table, including the length of ORF, the start and end positions of the location, et al. (C) The genome location, protein coding potential of CERS6-AS1 was further analyzed by using the PhyloCSF track. All values of phyloCSF were less than 0, indicating that CERS6-AS1 did not have coded conservative types.

**Supplementary Figure 2. The expression difference of CERS6-AS1, miR-30b-3p, MDM2 in pancreatic cancer tissues.** (A) CERS6-AS1 expression in different tumor grade. (B) CERS6-AS1 expression in different tumor stage. (C) miR-30b-3p expression in tumor and normal tissues. (D) miR-30b-3p expression in different tumor stage. (E) miR-30b-3p expression in different tumor grade. (F) MDM2 expression in tumor and normal tissues. (G) MDM2 expression in different tumor stage. (H) MDM2 expression in different tumor grade.

**Supplementary Figure 3. The statistical graph of relative protein expression**. (A) The statistical graph of Figure 2L. (B) The statistical graph of Figure 2M. (C) The statistical graph of Figure 3I. (D) The statistical graph of Figure 3J. (E) The statistical graph of Figure 6J. (F) The statistical graph of Figure 7D.
