# Supplementary material for: Long noncoding RNA CERS6-AS1 modulates glucose metabolism and tumor progression in hepatocellular carcinoma by promoting the MDM2/p53 signaling pathway
Source: Cell Death Discov. 2022 Aug 4;8:348. doi: 10.1038/s41420-022-01150-x (PMC9352870; doi:10.1038/s41420-022-01150-x)
Supplement: Supplementary file 2 — Supplemental Table 1 [file 41420_2022_1150_MOESM2_ESM.docx]

**Supplemental Table 1. The sequences of shRNA in this study**

| Name | Sequence (5’-3’) |
| --- | --- |
| sh-CERS6-AS1-1  Target (586-608) | CCGGTTTTTTAGCTATTGATTTCCCCTCGAGGAAATCAATAGCTAAAAAATGTTTTTG |
| sh-CERS6-AS1-2  Target (2758-2780) | CCGGTTTGTAAATTCAACTATAGGTCTCGAGCTATAGTTGAATTTACAAAATTTTTTG |
| sh-NC | CCGGTAGATCACGAGACACTCCGACTCGAGTCGGACGTGTTTGCTGATCTTTTTTG |
