# Supplementary figures and images for: Long noncoding RNA CERS6-AS1 modulates glucose metabolism and tumor progression in hepatocellular carcinoma by promoting the MDM2/p53 signaling pathway
Source: Cell Death Discov. 2022 Aug 4;8:348. doi: 10.1038/s41420-022-01150-x (PMC9352870; doi:10.1038/s41420-022-01150-x)

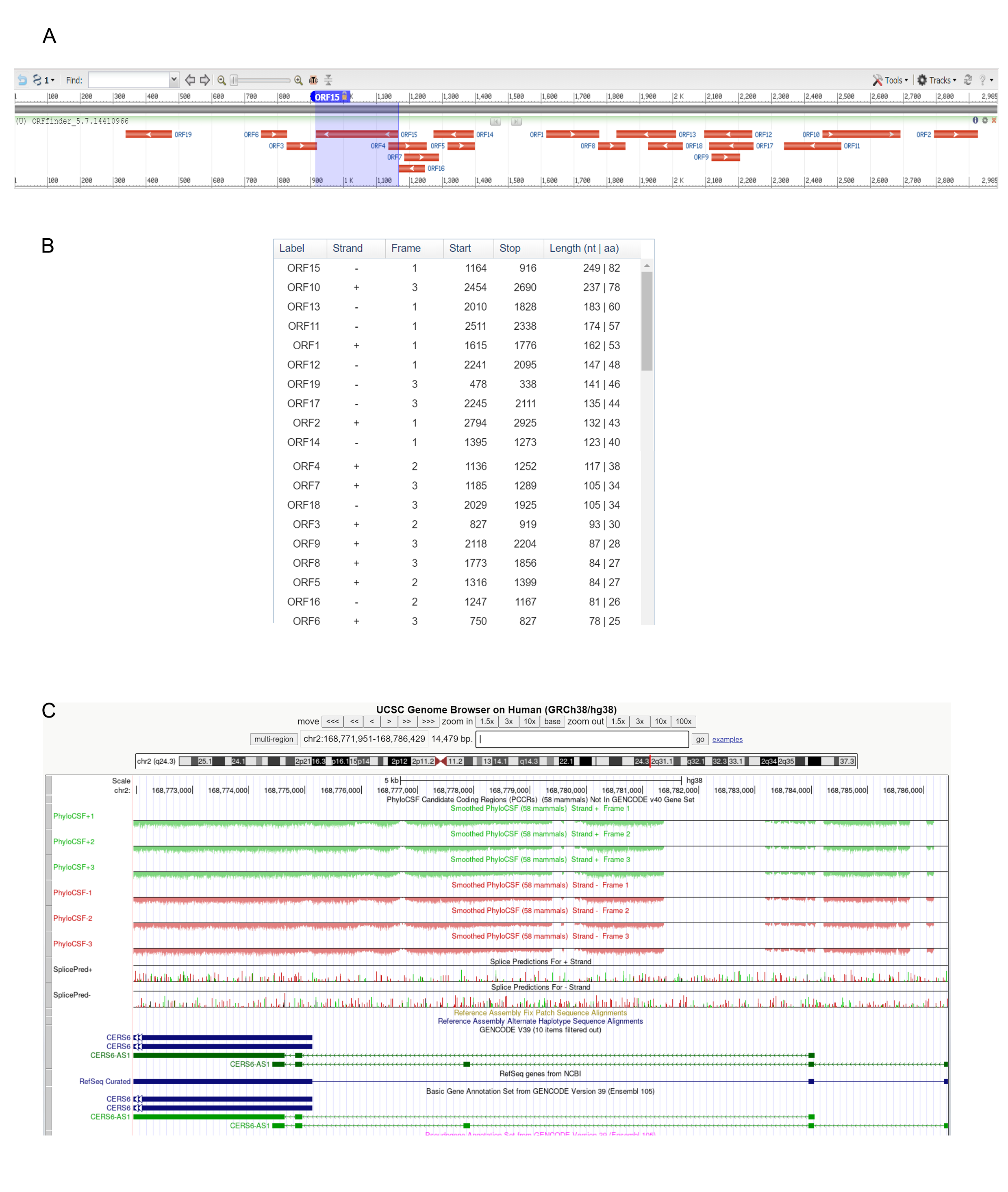

Supplement: Supplementary file 3 — supplemental Figure 1 [file 41420_2022_1150_MOESM3_ESM.tif]

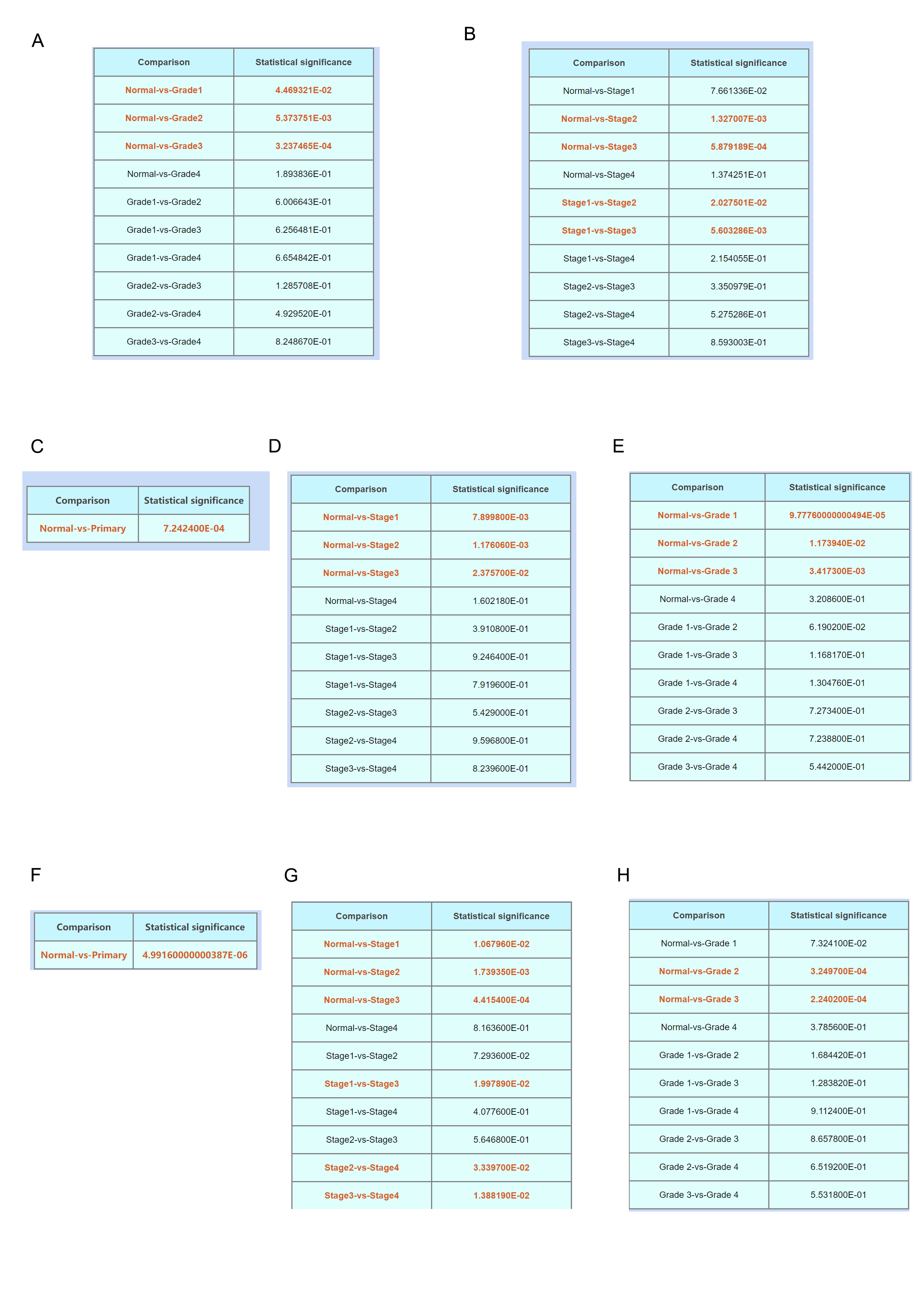

Supplement: Supplementary file 4 — supplemental Figure 2 [file 41420_2022_1150_MOESM4_ESM.tif]

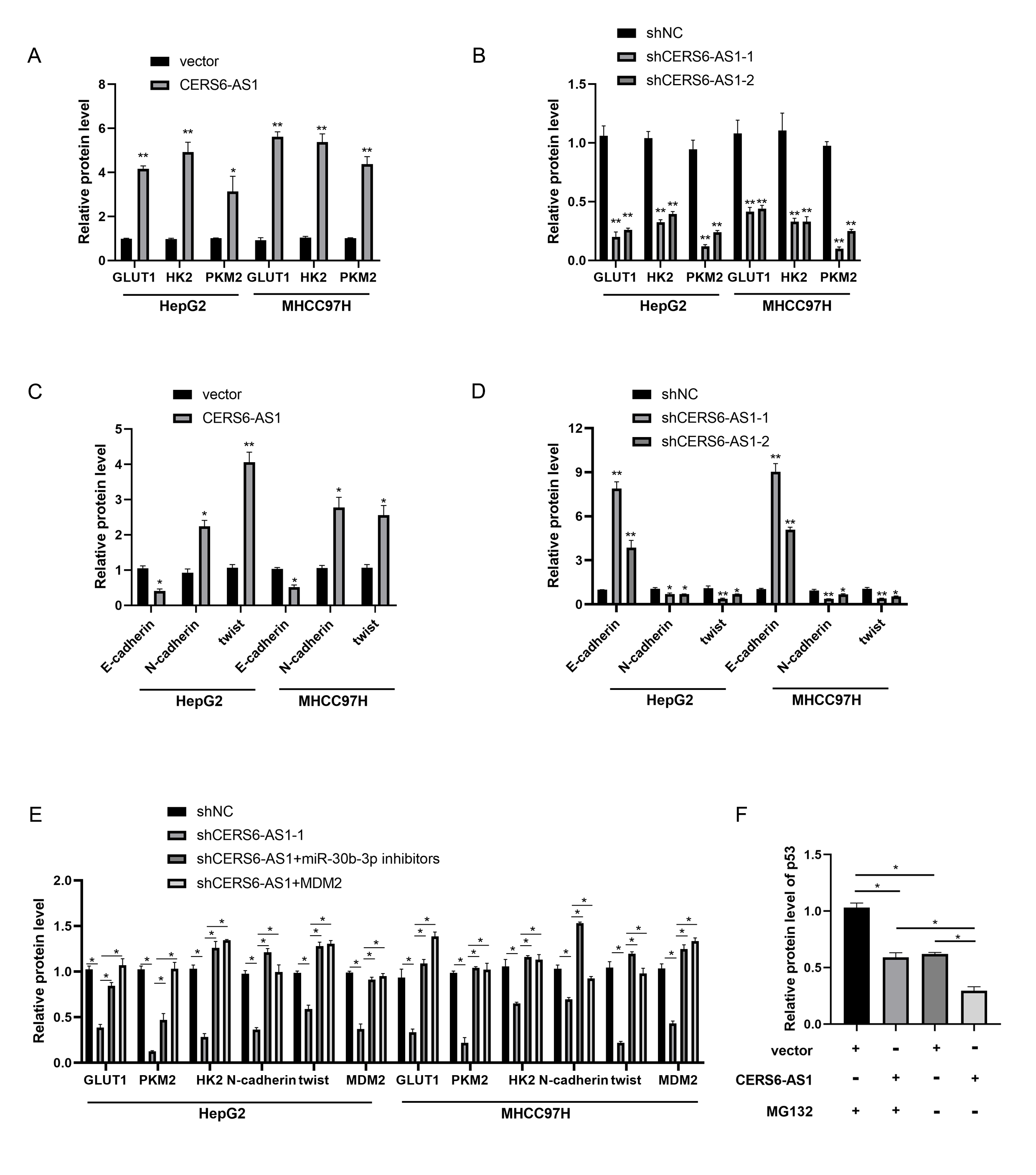

Supplement: Supplementary file 5 — supplemental Figure 3 [file 41420_2022_1150_MOESM5_ESM.tif]
