# Supplementary material for: Long noncoding RNA CERS6-AS1 modulates glucose metabolism and tumor progression in hepatocellular carcinoma by promoting the MDM2/p53 signaling pathway
Source: Cell Death Discov. 2022 Aug 4;8:348. doi: 10.1038/s41420-022-01150-x (PMC9352870; doi:10.1038/s41420-022-01150-x)

Figure 6J

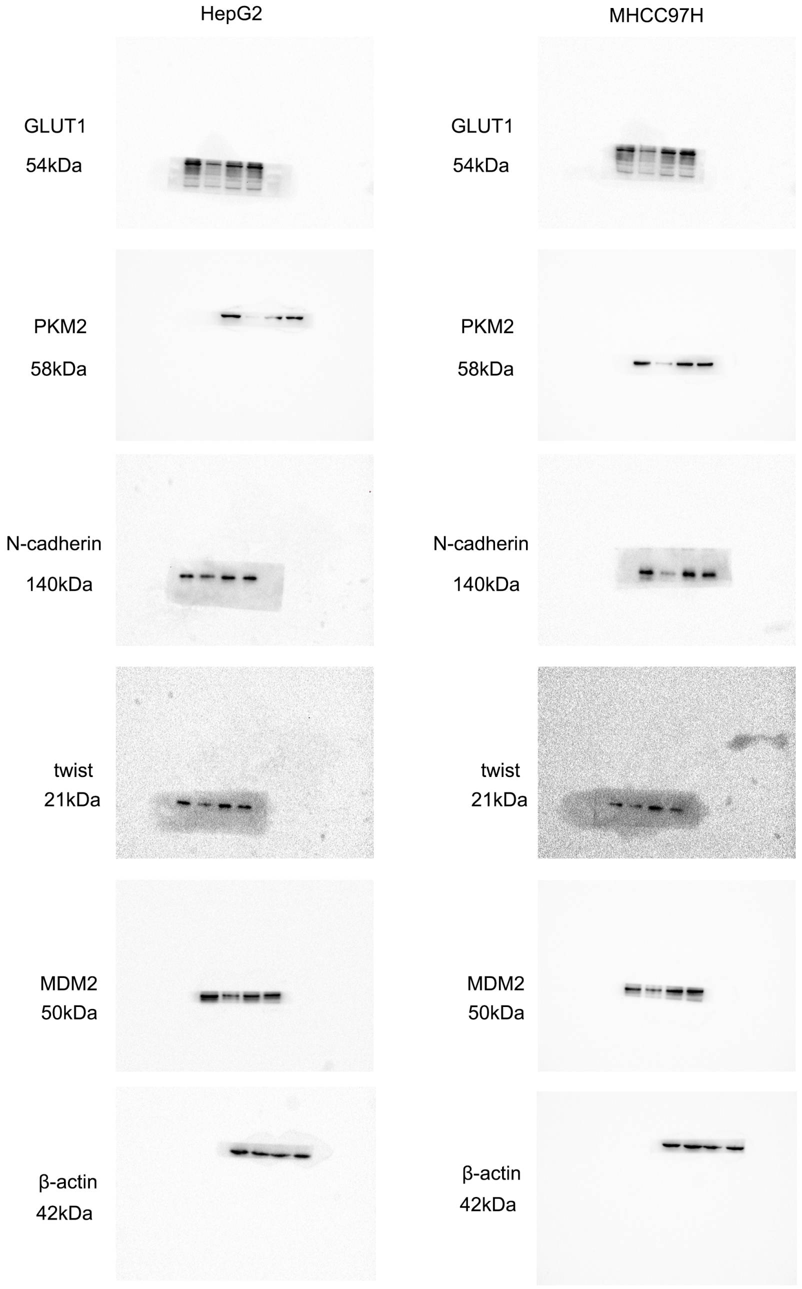
HepG2
MHCC97H
GLUT1
54kDa
GLUT1
54kDa
PKM2
58kDa
PKM2
58kDa
N-cadherin
140kDa
N-cadherin
140kDa
twist
21kDa
twist
21kDa
MDM2
50kDa
MDM2
50kDa
β-actin
42kDa
β-actin
42kDa

Figure 7C

MDM2
50kDa

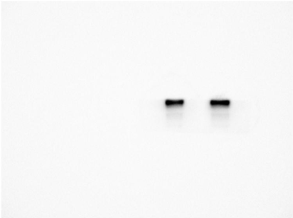

p53
53kDa

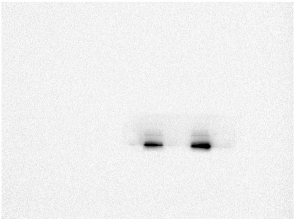

Figure 7D

p53
53kDa

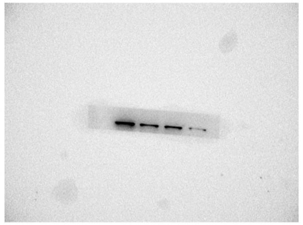

GAPDH
36kDa

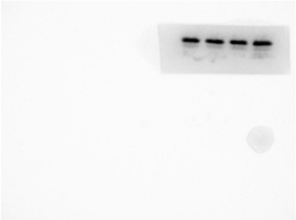

Figure 7E

P53
53kDa

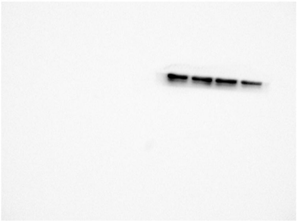

p53
53kDa

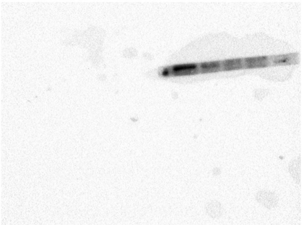

GAPDH
36kDa

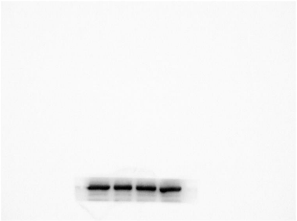

GAPDH
36kDa

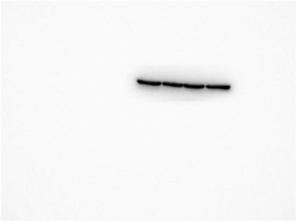

Figure 7G

IP:Flag

25-170kDa

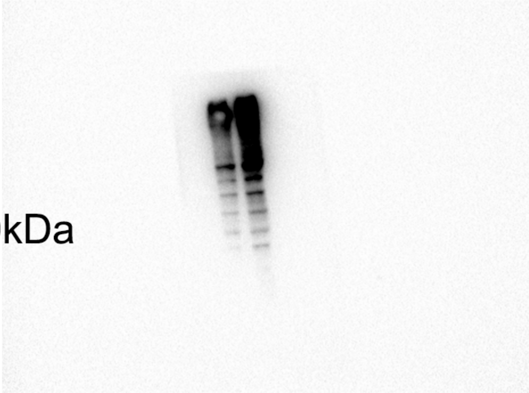

IB:Flag

53kDa

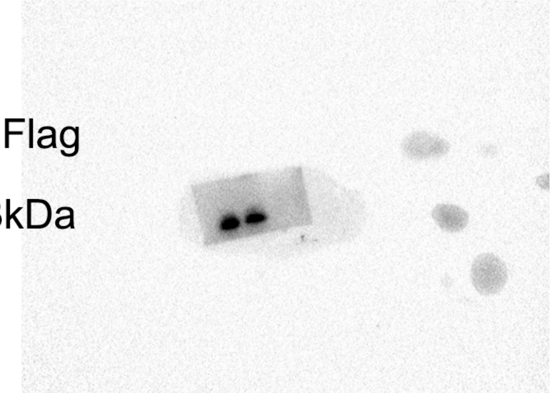

MDM2

50kDa

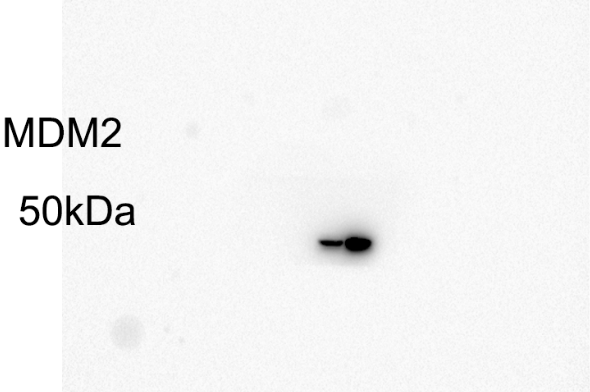

Flag

53kDa

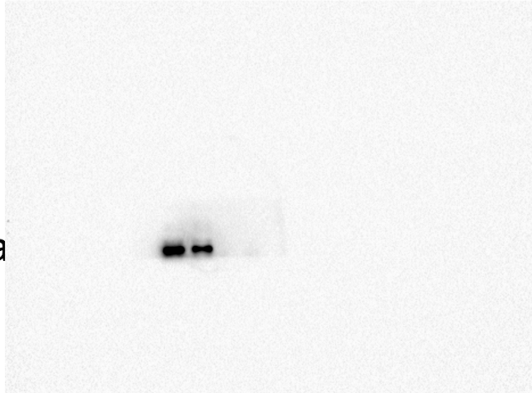

GAPDH

36kDa

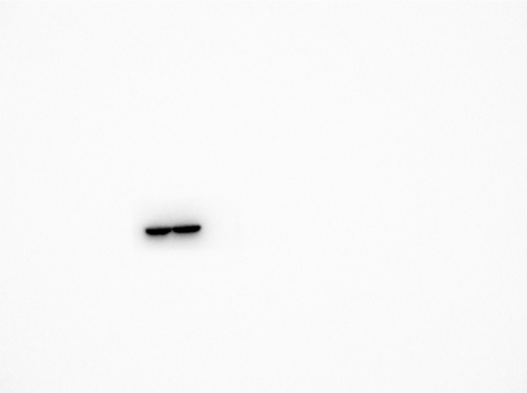

Figure 7H

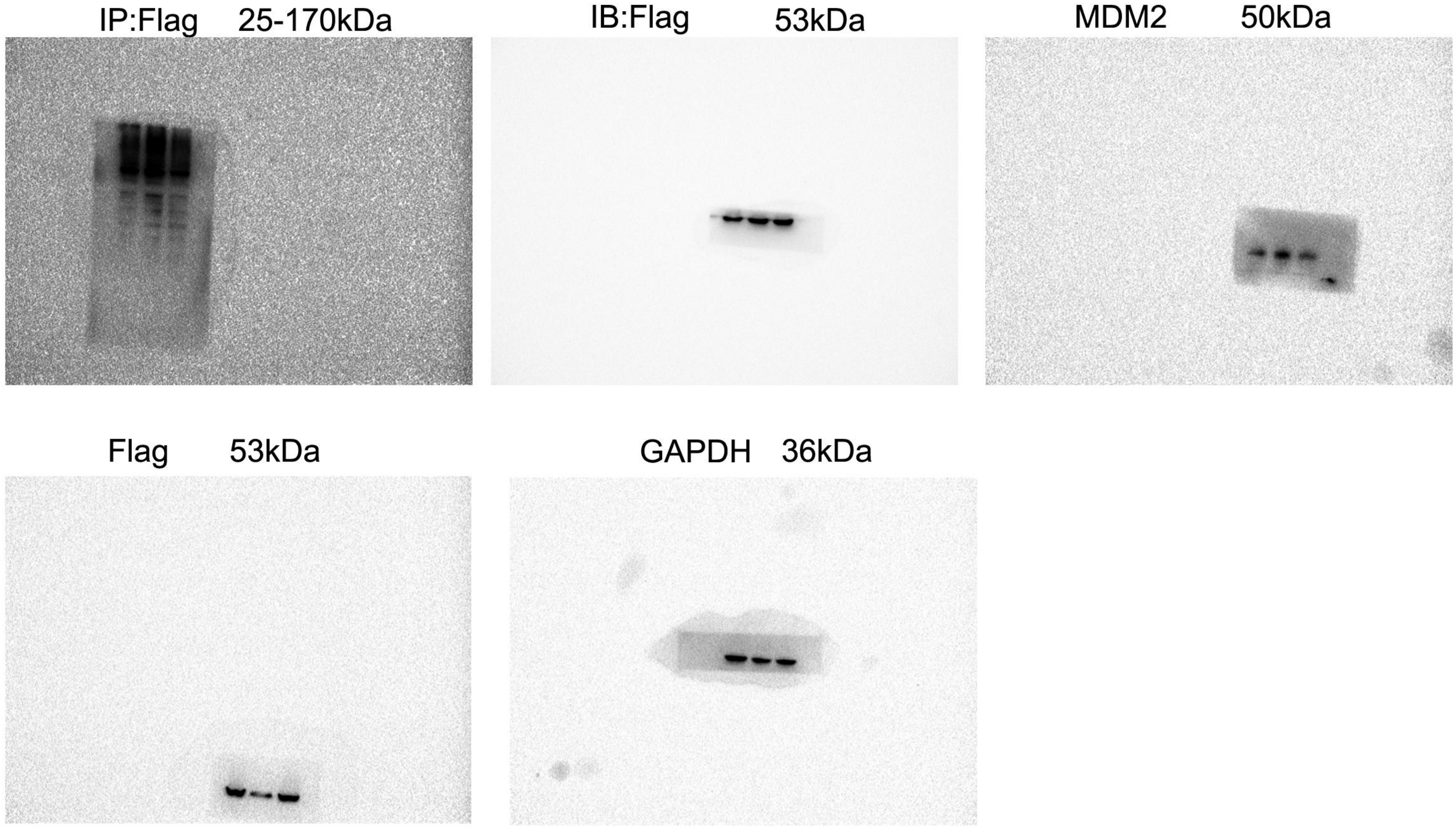
IP:Flag 25-170kDa
IB:Flag 53kDa
MDM2 50kDa
Flag 53kDa
GAPDH 36kDa

Figure 2L

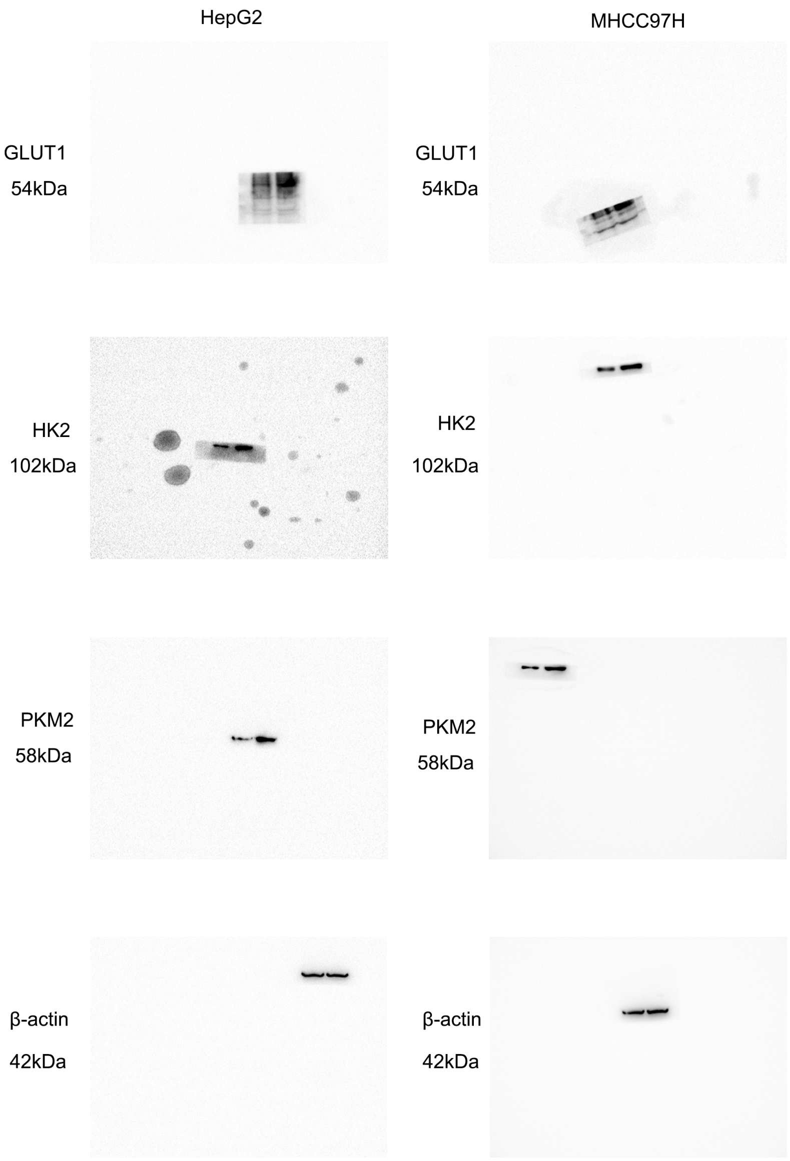

HepG2
MHCC97H
GLUT1
54kDa
GLUT1
54kDa
HK2
102kDa
HK2
102kDa
PKM2
58kDa
PKM2
58kDa
β-actin
42kDa
β-actin
42kDa

Figure 2M

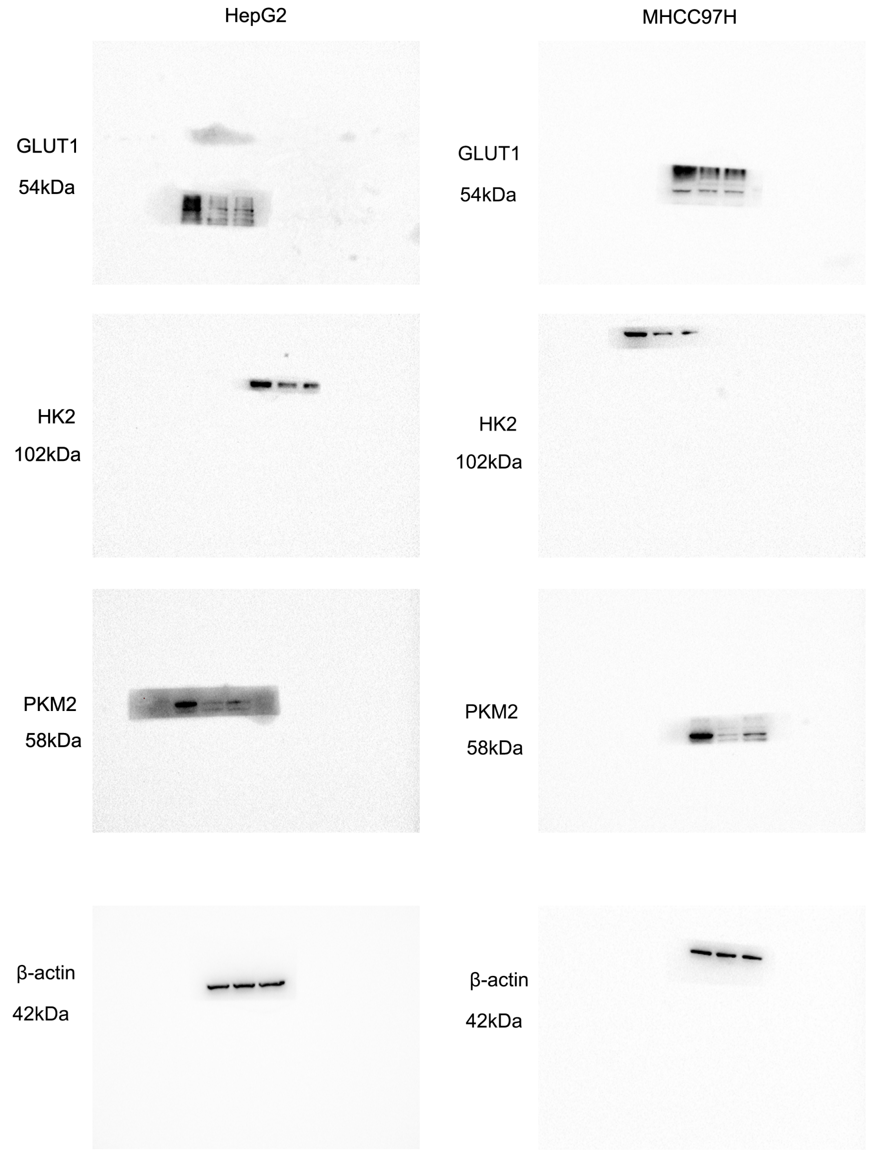
HepG2
MHCC97H
GLUT1
54kDa
GLUT1
54kDa
HK2
102kDa
HK2
102kDa
PKM2
58kDa
PKM2
58kDa
β-actin
42kDa
β-actin
42kDa

Figure 3I

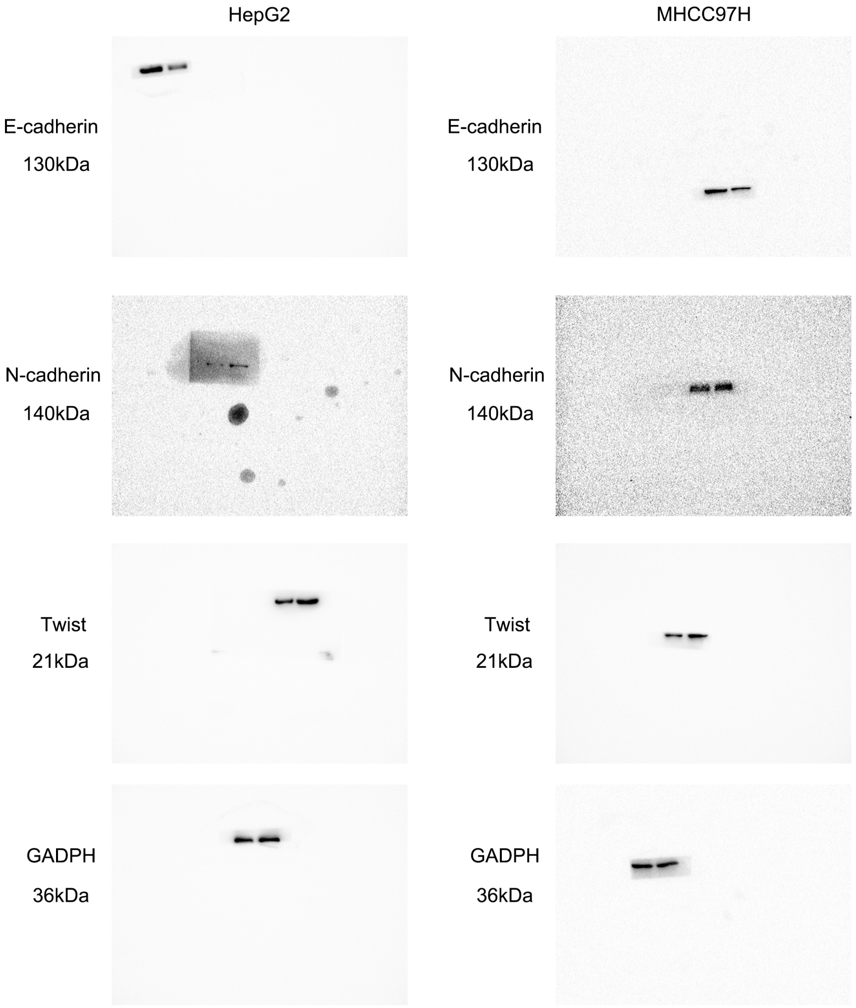
HepG2
MHCC97H
E-cadherin
130kDa
E-cadherin
130kDa
N-cadherin
140kDa
N-cadherin
140kDa
Twist
21kDa
Twist
21kDa
GADPH
36kDa
GADPH
36kDa

Figure 3J

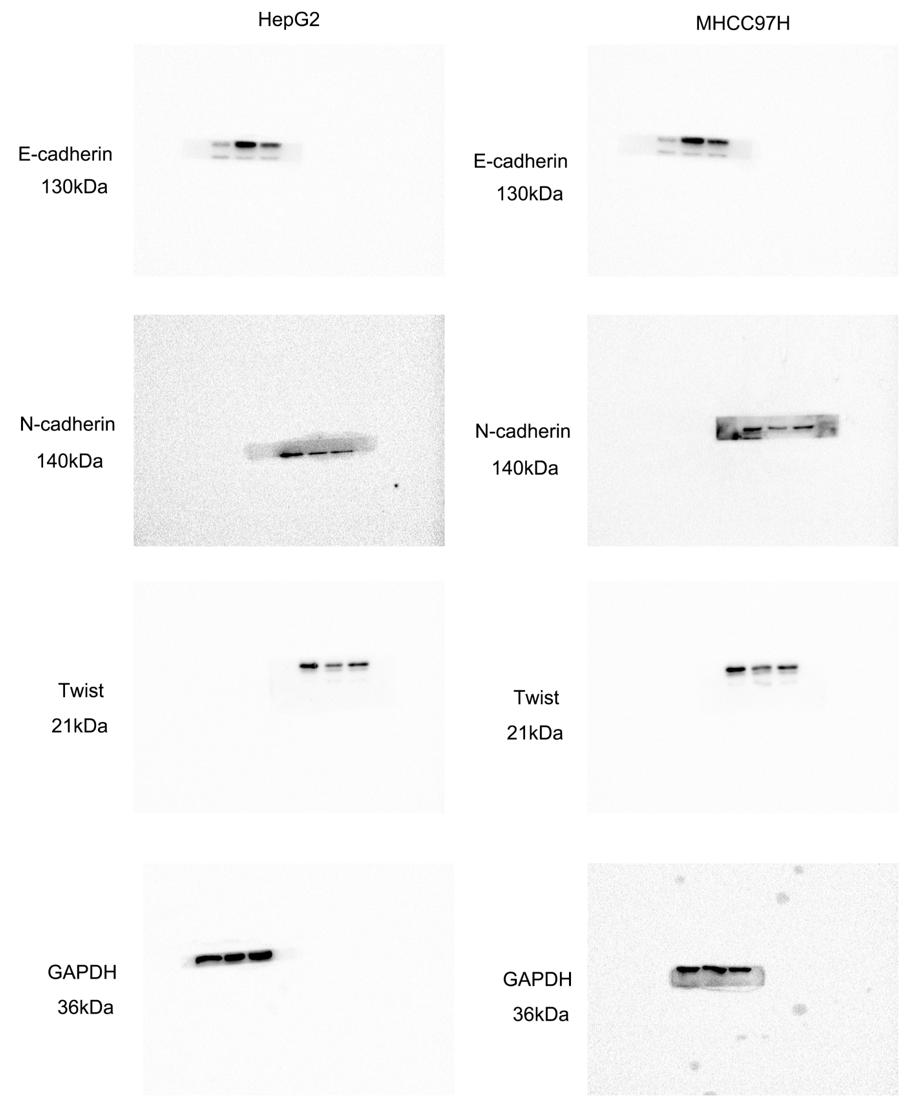
HepG2
MHCC97H
E-cadherin
130kDa
E-cadherin
130kDa
N-cadherin
140kDa
N-cadherin
140kDa
Twist
21kDa
Twist
21kDa
GAPDH
36kDa
GAPDH
36kDa

Figure 5F

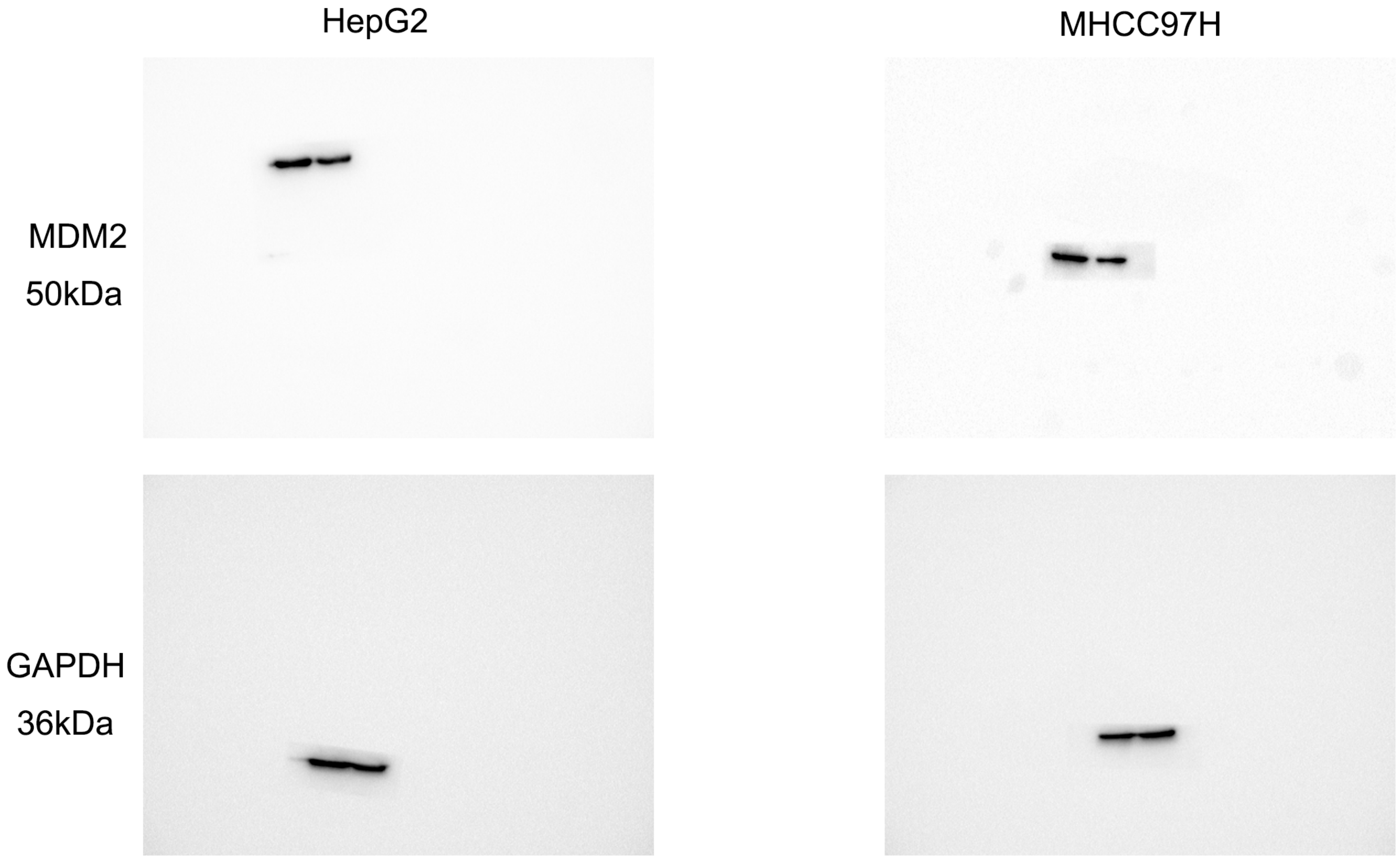

Supplement: Supplementary file 6 — Original Data File [file 41420_2022_1150_MOESM6_ESM.pdf]
