# Supplementary material for: Long noncoding RNA CERS6-AS1 modulates glucose metabolism and tumor progression in hepatocellular carcinoma by promoting the MDM2/p53 signaling pathway
Source: Cell Death Discov. 2022 Aug 4;8:348. doi: 10.1038/s41420-022-01150-x (PMC9352870; doi:10.1038/s41420-022-01150-x)
Supplement: Supplementary file 7 — English editing Certificate [file 41420_2022_1150_MOESM7_ESM.pdf]

edanz 理文编辑
INNOVATIVE SCIENTIFIC SOLUTIONS

Certificate of Editing

Edited provisional title

Long noncoding RNA CERS6-AS1 modulates glucose metabolism and tumor progression in hepatocellular carcinoma by promoting the MDM2/p53 signaling pathway

Client name and institution

Bo Xu, Department of Liver Surgery & Liver Transplantation Center, West China Hospital of Sichuan University, Chengdu, Sichuan, China.

Date Completed
2021-12-24

Identification code
101232

Certificate issued by
Koji Yamashita
Managing Director and CEO

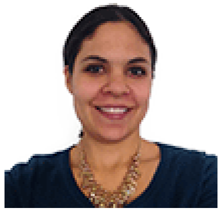

Expert Editor: Gabrielle White Wolf
2005 PhD Genetics and Molecular Biology
University of North Carolina at Chapel Hill
Genetics, Medical Biochemistry and Metabolomics, Oncology and Carcinogenesis

www.liwenbianji.cn

While this certificate confirms the authors have used Edanz's editing services, we cannot guarantee that additional changes have not been made after our edits.
